# Evolutionary Analysis Predicts Sensitive Positions of MMP20 and Validates Newly- and Previously-Identified *MMP20* Mutations Causing Amelogenesis Imperfecta

**DOI:** 10.3389/fphys.2017.00398

**Published:** 2017-06-14

**Authors:** Barbara Gasse, Megana Prasad, Sidney Delgado, Mathilde Huckert, Marzena Kawczynski, Annelyse Garret-Bernardin, Serena Lopez-Cazaux, Isabelle Bailleul-Forestier, Marie-Cécile Manière, Corinne Stoetzel, Agnès Bloch-Zupan, Jean-Yves Sire

**Affiliations:** ^1^Institut de Biologie Paris-Seine, UMR 7138-Evolution Paris-Seine, Sorbonne Universités, Université Pierre et Marie CurieParis, France; ^2^Laboratoire de Génétique Médicale, Institut National de la Santé et de la Recherche Médicale UMRS_1112, Institut de Génétique Médicale d'Alsace, FMTS, Université de StrasbourgStrasbourg, France; ^3^Faculté de Chirurgie Dentaire, Université de StrasbourgStrasbourg, France; ^4^Pôle de Médecine et Chirurgie Bucco-Dentaires, Centre de Référence des Manifestations Odontologiques des Maladies Rares, O-Rares, Hôpitaux Universitaires de StrasbourgStrasbourg, France; ^5^Unit of Dentistry, IRCCS, Bambino Gesù Children's HospitalRome, Italy; ^6^Faculté de Chirurgie Dentaire, Département d'Odontologie Pédiatrique, Centre de Compétences Maladies Rares, CHU Hôtel Dieu, Service d'odontologie Conservatrice et PédiatriqueNantes, France; ^7^Faculté de Chirurgie Dentaire, CHU de Toulouse, Centre de Compétences Maladies Rares, Odontologie Pédiatrique, Université Paul SabatierToulouse, France; ^8^Centre Européen de Recherche en Biologie et en Médecine, Centre National de la Recherche Scientifique UMR7104, Institut National de la Santé et de la Recherche Médicale U964, Institut de Génétique et de Biologie Moléculaire and Cellulaire, Université de StrasbourgIllkirch, France; ^9^Institut d'Etudes Avancées, Université de Strasbourg, USIASStrasbourg, France; ^10^Eastman Dental Institute, University College LondonLondon, United Kingdom

**Keywords:** MMP20, mutations, amelogenesis imperfecta, evolution, phenotype

## Abstract

Amelogenesis imperfecta (AI) designates a group of genetic diseases characterized by a large range of enamel disorders causing important social and health problems. These defects can result from mutations in enamel matrix proteins or protease encoding genes. A range of mutations in the enamel cleavage enzyme matrix metalloproteinase-20 gene (*MMP20*) produce enamel defects of varying severity. To address how various alterations produce a range of AI phenotypes, we performed a targeted analysis to find *MMP20* mutations in French patients diagnosed with non-syndromic AI. Genomic DNA was isolated from saliva and *MMP20* exons and exon-intron boundaries sequenced. We identified several homozygous or heterozygous mutations, putatively involved in the AI phenotypes. To validate missense mutations and predict sensitive positions in the MMP20 sequence, we evolutionarily compared 75 sequences extracted from the public databases using the Datamonkey webserver. These sequences were representative of mammalian lineages, covering more than 150 million years of evolution. This analysis allowed us to find 324 sensitive positions (out of the 483 MMP20 residues), pinpoint functionally important domains, and build an evolutionary chart of important conserved MMP20 regions. This is an efficient tool to identify new- and previously-identified mutations. We thus identified six functional *MMP20* mutations in unrelated families, finding two novel mutated sites. The genotypes and phenotypes of these six mutations are described and compared. To date, 13 *MMP20* mutations causing AI have been reported, making these genotypes and associated hypomature enamel phenotypes the most frequent in AI.

## Introduction

Amelogenesis imperfecta (AI) describes a group of genetic diseases producing a large range of alterations in enamel structure. Defects include hypoplastic, hypomineralized, or hypomature enamel structure and altered enamel appearance -including rough, pitted, banded, or discolored teeth. These alterations produce severe health problems and impair normal social interactions. Defects can result from mutations either of enamel matrix protein encoding genes [amelogenin (*AMELX*), ameloblastin (*AMBN*), amelotin (*AMTN*), and enamelin (*ENAM*)], or proteases [matrix metalloproteinase-20 (*MMP20*), kallikrein-related peptidase 4 (*KLK4*))]—often displaying specific roles in tooth—and hence at the current stage of our knowledge do not display disorders elsewhere in the body. Other genes encoding proteins having functions in cell attachment, ionic transport, and mineralization processes, when mutated are responsible for AI phenotypes associated with various abnormalities in syndromes (Crawford et al., 2007; reviewed in Bloch-Zupan et al., 2012).

The prevalence of AI can vary from 1:700 to 1:14,000 depending on the country. Although many candidate genes were identified to date, many AI-producing mutations remain to be identified for approximately 50% of diagnosed patients.

Here, we focused on the matrix metalloproteinase-20 gene (*MMP20*), an interesting candidate gene given the numerous identified AI causing mutations (Kim et al., 2005, 2016a; Ozdemir et al., 2005; Papagerakis et al., 2008; Lee et al., 2010; Gasse et al., 2013; Wang et al., 2013; Seymen et al., 2015; Prasad et al., 2016a).

MMPs function in plants, invertebrates, and vertebrates, by their active center possessing a catalytic zinc domain (Gomis-Ruth, 2009; Fanjul-Fernandez et al., 2010). In vertebrates, matrix metalloproteinases (MMPs, also called matrixins) consist of a large family (24 members identified in humans) of endopeptidases, which are phylogenetically related (Fanjul-Fernandez et al., 2010). MMPs were initially thought involved in the degradation and turnover of the extracellular matrix, but recent studies indicate important biological roles regulating cell behavior and signaling pathways (Rodríguez et al., 2010).

MMP20 (also termed enamelysin) is only present in vertebrates. Its origin dates certainly back to an ancestral gnathostome, before the divergence of actinopterygians and sarcopterygians more than 450 million years ago (Kawasaki and Suzuki, 2011). In amniotes, MMP20 is an enamel specific protease. This gene is absent in every species lacking either teeth or enamel (e.g., in turtles, birds, and various mammals -Meredith et al., 2011, 2013).

In toothed mammals, MMP20 was identified first in porcine enamel (Bartlett et al., 1996; Moradian-Oldak et al., 1996) then characterized in humans (Llano et al., 1997). It is expressed both by ameloblasts and odontoblasts, and acts from the enamel secretory to the maturation stages through proteolysis of the enamel organic matrix, required for correct mineralization (Bègue-Kirn et al., 1998). MMP20 cleaves AMEL (Llano et al., 1997; Ryu et al., 1999; Nagano et al., 2009), the enamel matrix protein (EMP) representing more than 90% of the forming enamel matrix in mammals (Fincham et al., 1989). MMP20 also cleaves AMBN, and probably also ENAM, two EMPs with critical functions during enamel mineralization (Iwata et al., 2007; Chun et al., 2010).

Mutations in the *MMP20* gene have been associated with autosomal recessive type 2 amelogenesis imperfecta (AI2A2, MIM #612529, ORPHA100033) also called hypomature AI. In affected patients, enamel displays a normal thickness but is pigmented and hypomineralized as demonstrated by the lack of radio-opacity contrast with dentin (Witkop, 1989).

To find mutations on *MMP20* in French patients diagnosed for AI, we sequenced and identified several new homozygous or heterozygous missense mutations. To validate the potential role of these mutations, hence to predict sensitive positions in MMP20, we analyzed a large set of representative mammalian lineages sequences, covering over 150 million years of evolution. This type of evolutionary analysis has been shown to be an efficient method to validate and predict disease-associated missense mutations (Delgado et al., 2007; Al Hashimi et al., 2009; Bardet et al., 2010; Silvent et al., 2014). This method is termed phylomedicine (Kumar et al., 2011), which is complementary to existing genetic diagnosis.

The aims of the present study were to document MMP20 evolutionary analysis to pinpoint where newly identified mutations act in this evolutionary chart, hence identifying sensitive positions. Collectively this is an efficient tool to functionally validate *MMP20* mutations identified to date.

## Materials and methods

### Evolutionary analysis

#### Data set

Mammalian *MMP20* sequences were extracted from public databases, *NCBI* [http://www.ncbi.nlm.nih.gov] and *Ensembl* [http://www.ensembl.org]. A total of 75 sequences representative of the main mammalian lineages (55 families distributed within 19 orders) were retained for our analyses (Supplementary Table 1). Identical sequences (such as species from the same genus) were not included in our dataset. *MMP20* being enamel specific the sequences of species lacking either teeth or enamel (i.e., Xenarthra, Pholidota, Mysticeta, and Tubulidentata) were not included in our study because they display various mutations.

Among the selected *MMP20* sequences only five were published in GenBank. Of the 70 other sequences, 65 were computer-predicted from sequenced genomes and six were obtained using Basic Local Alignment Search Tool (BLAST) of the whole genome shotgun (WGS) repository sequences in *NCBI* (Supplementary Table 1). The coding sequences were traduced into amino acid sequences and unpublished sequences were validated through alignment to published ones using *Se-Al v.2.0a11* software [http://tree.bio.ed.ac.uk/software/seal]. The intron-exon boundaries were also carefully checked. The dataset of the 75 MMP20 sequences is available in Supplementary Data 1.

Our final alignment consisted of 483 positions, and no insertions were needed (Supplementary Figure 1). A few residues were missing in some, uncompletely sequenced genomic DNA (i.e., 726 nucleotides, nt, representing <0.7% of the data), and the corresponding positions were treated as “unknown data”. In addition, we aligned 21 nucleotides of the intronic region located on both sides of the exons from 12 *MMP20* sequences representative of the main mammalian lineages, known to be important for correct intron splicing (Supplementary Table 2).

#### Analyses

The putative signal peptide sequence and its cleavage site were predicted using *SignalP 3.0 server* (http://www.cbs.dtu.dk/services/SignalP).

Single Likelihood Ancestor Counting (SLAC) analysis was performed using the Datamonkey webserver (http://www.datamonkey.org/; Delport et al., 2010) to identify amino acids subjected either to purifying or to positive selection, as previously described (Silvent et al., 2014). Biologically significant amino acids (i.e., site-specific selections) in MMP20 were identified in our alignment for the 483 positions and displayed on the human sequence. The analysis was performed according to the substitution preferences of amino acids, i.e., favoring property conservation (see Silvent et al., 2014). We defined three levels of selection throughout mammalian evolution (i.e., 180 Ma): conserved (i.e., unchanged residues), conservative (i.e., substituted residues having similar properties) and variable positions (i.e., substitution with various residues).

### Mutation analyses

#### Patients

The patients and their families were selected from the pool of patients participating in the French Ministry of Health National Program for Clinical Research, PHRC 2008 HUS (Strasbourg University Hospital) N°4266, Amelogenesis Imperfecta, AI (for further details see Gasse et al., 2013) and in the INTERREG IV Offensive Sciences A27 “Orodental manifestation of rare diseases” EU funded (ERDF) project.

These patients came to The Reference Centre for Orodental Manifestations of Rare Diseases (CRMR Strasbourg, France) or other affiliated Competence Centres (CCMR) for clinical diagnosis and management. Dentists specializing in rare diseases diagnosed amelogenesis imperfecta.

The oral phenotypes were documented using the D[4]/phenodent registry, a Diagnosing Dental Defects Database [see www.phenodent.org, to access assessment form], which is approved by CNIL (French National commission for informatics and liberty, number 908416). This clinical study is registered at https://clinicaltrials.gov: NCT01746121 and NCT02397824, and with the MESR (French Ministry of Higher Education and Research) Bioethics Commission as a biological collection “Orodental Manifestations of Rare Diseases” DC-2012-1677 within DC-2012-1002 and was acknowledged by the CPP (person protection committee) Est IV on the 11 Dec 2012.

Affected and unaffected family members gave informed written consents both for the D4/phenodent registry and for genetic analyses performed on the salivary samples included in the biological collection.

In this study, we selected patients from unrelated families suffering from non-syndromic AI, some of them displaying clinical diagnoses matching possible *MMP20* mutations.

#### Analyses

Patients spit into an Oragene kit (Oragene DNA®, DNA Genotek, Canada) and genomic DNA was then isolated from saliva according to the manufacturer's protocol. We used previously defined primers (see Gasse et al., 2013). Mutational analysis was performed for the 10 exons of *MMP20* including exon-intron boundaries. PCR products were sent to GATC Biotech for purification and sequencing in both directions in order to minimize sequencing artifacts. The sequences were aligned manually with the reference human *MMP20* sequence NG_012151.1 using *Se-Al v2.0a11* software.

When necessary the sequences were analyzed for splicing site prediction using the NetGene2 server (http://www.cbs.dtu.dk/services/NetGene2/) and MaxEntScan (http://genes.mit.edu/burgelab/maxent/Xmaxentscan_scoreseq.html). The NetGene2 server is a service producing neural network predictions of splice sites in human genes (Hebsgaard et al., 1996) and MaxEntScan is based on the “Maximum Entropy Principle” and generalizes most previous probabilistic models of sequence motifs such as weight matrix models and inhomogeneous Markov models (Yeo and Burge, 2004).

SNPs known to date in the human *MMP20* sequence were found at http://www.ncbi.nlm.nih.gov/snp and at https://www.ncbi.nlm.nih.gov/variation/tools/1000 genomes/(the 1000 genomes project).

## Results and discussion

### Evolutionary analysis of functional constraints in MMP20 sequence

The alignment of the 75 MMP20 amino acid (aa) sequences indicated a highly conserved protein structure throughout more than 180 million years of mammalian evolution, a finding demonstrating the importance of many regions of this protease as found for the alkaline phosphatase, ALPL (Silvent et al., 2014). The 10 exons encoding the protein do not show insertions and only a limited number of rodent-specific deletions, in which seven MMP20 lack either one codon in exon 1 (*Mus, Rattus, Cricetulus, Mesocricetus, Microtus*, and *Chinchilla*) or two codons in exon 2 (*Octodon*). The MMP20 sequences were therefore mostly composed of 483 residues, from the methionine M^1^, encoded by exon 1, to the cysteine C^483^ encoded by exon 10 and preceding the stop codon (Supplementary Figure 1). At the first glance our alignment indicated that many positions and large domains were conserved throughout the sequence. This is particularly obvious in the large regions encoded by the 3' end of exon 2, by exons 4 and 5, by the 3′ region of exon 6, by most of exons 7 and 8, and by many positions of exons 9 and 10 (Supplementary Figure 1). The alignment of the 21 untranslated nucleotides on both sides of the coding exons indicates that the acceptor sites are either tag or cag, and the donor sites are mostly gta (with the exception of gtt for intron 1 and gtg for intron 9 and in a few other sequences; Supplementary Table 2). This finding is in accordance with the splice site consensus sequences for introns. The other positions are somewhat variable with the exception of the 21 nucleotides of the 5′ region of intron 4 that are unchanged in all species studied (i.e., for 180 MA). We do not know why this region was unchanged during evolution but its conservation suggests functionally important domains otherwise some nucleotide substitutions would have occurred at random. We question whether this intron region could be involved in a regulatory process or was previously encoding region.

**Figure 1:**
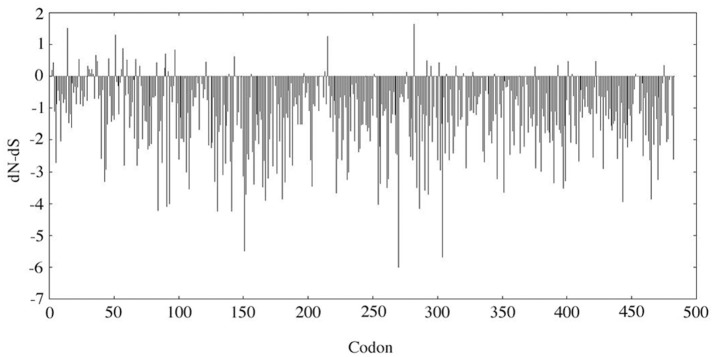
SLAC analysis. The dN-dS value was analyzed along the MMP20 codon sequence. dN, non-synonymous substitution rate; dS, synonymous substitution rate. When dN-dS < 0, the codon is subjected to purifying selection. When dN-dS > 0 (i.e., dN > dS), the codon is considered positively selected.

**Figure 2:**
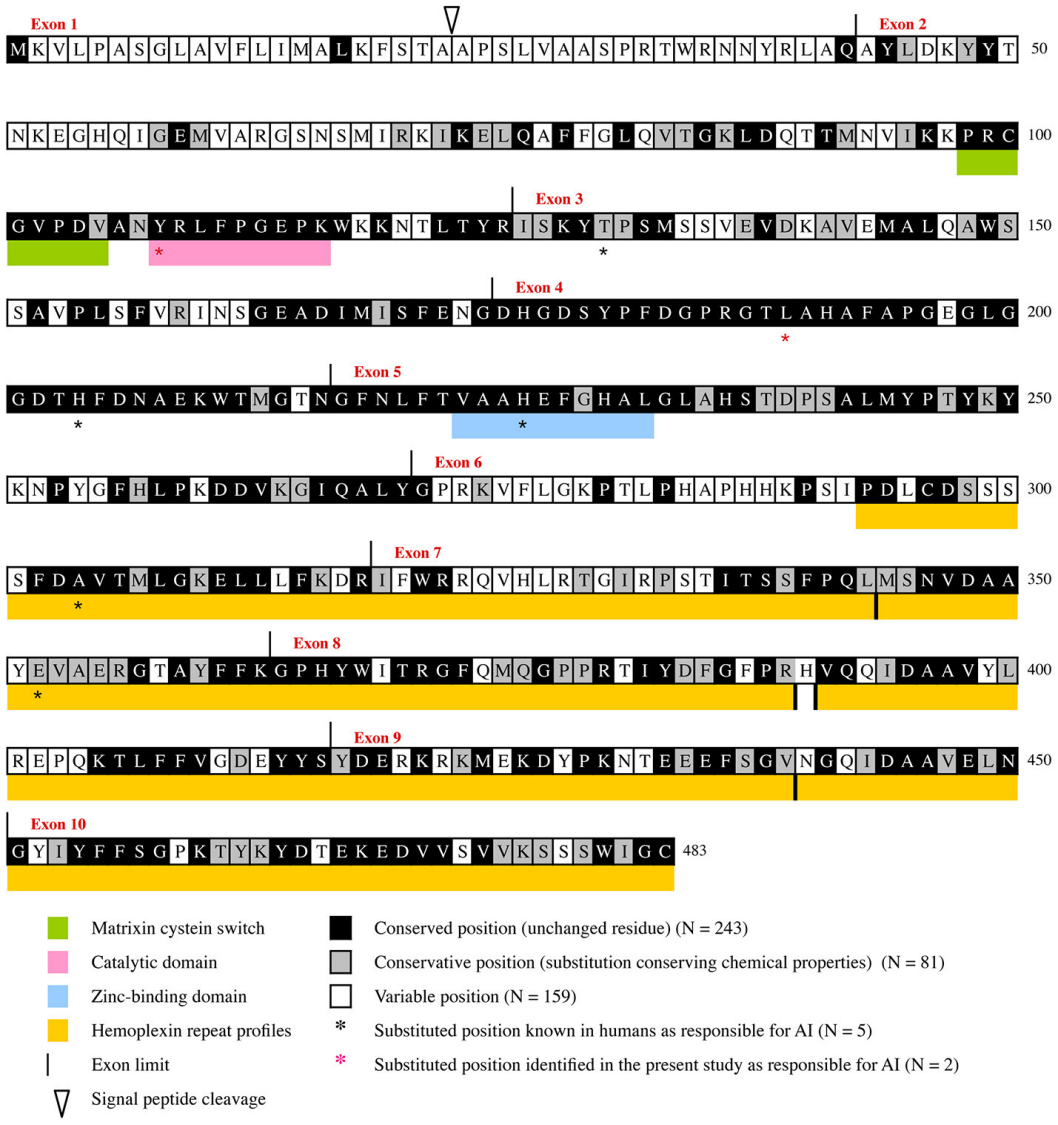
Evolutionary chart of MMP20. This chart was obtained from the alignment of 75 sequences representative of the main mammalian lineages (approx. 180 million years of evolution) and was deduced from the results obtained when dN/dS was calculated at each codon of MMP20 by Consurf (see Supplementary Figures 1, 2). Human sequence is used as a reference. Positions subjected to purifying selecton are on black (conserved positions) and gray (conservative positions) background. Variable positions on white background.

The evolutionary analysis using SLAC selected several positions, notably in the N-terminal region, and, in contrast indicated that the MMP20 sequence is globally under strong purifying selection (Figure 1). The detailed analysis of each position confirmed the numerous fonctional or structural constraints acting on many amino acids along the sequence (Figure 2, Supplementary Figure 2). Out of the 483 amino acids composing the human MMP20 sequence, more than a half (324 aa, 67.08%) were identified as sensitive positions, i.e., that were either conserved (i.e., unchanged residues) (243 aa, 50.31%) or conservative (i.e., substituted with residues having similar properties) (81 aa, 16.77%) during 180 million years of mammalian evolution (Figure 2). In contrast, 159 positions (32.92%) were identified as variable (i.e., substituted with various aa). This large number of sensitive positions revealed by our analysis is similar to the values obtained for ALPL (Silvent et al., 2014). These unchanged, conservative and variable positions were reported on the human sequence, resulting in the chart of sensitive positions of human MMP20 (Figure 2, Supplementary Figure 2). We predict that any substitution of one of the 243 unchanged positions, or of one of the 81 conservative positions with a residue having a different property, would disrupt MMP20 function and would lead to enamel defects described as amelogenesis imperfecta in patients who unfortunately possess such a mutation in both DNA alleles (homozygous mutation) or an additional mutation in the other allele (counpound heterozygous mutation). Indeed, all missense mutations validated until now in various proteins were located always at conserved positions (Delgado et al., 2007; Kumar et al., 2011).

The boundaries of three, already known, functional domains are also better defined when considering the number of conserved amino acids: the matrixin cystein switch extends from aa98 to aa104, the catalytic domain from aa108 to aa116, and the zinc-binding domain from aa223 to aa232. Similar, accurate definition of the boundaries of functional domains was obtain in various proteins through evolutionary analysis (Al Hashimi et al., 2009; Silvent et al., 2013, 2014). Aside these crucial regions our study highlighted many other evolutionary-conserved residues and domains that have probably a strong functional or structural importance for MMP20. One of these large domains, encoded by the 3′ end of exon 3, exon 4, and most of exon 5, is composed of 109 residues, out of them 102 (93.58%) are either unchanged or conservative. Several, but shorter domains are also encoded by the 3′ region of exons 6 and 7, and by most of the sequence of exons 8–10 (Figure 2, Supplementary Figure 2). The only 70 aa located at the N-terminal region are subjected to low selective constraints with 15.71% of sensitive positions detected.

It is worth noting that the percentage of purifying selection is high along the protein sequence, and varies from 7.14% in the region encoded by exon 1–95.24% in the region encoded by exon 4. More precisely, our evolutionary analysis (i) confirmed and accurately defined the boundaries of already known important domains of the protein, (ii) highlighted many sensitive residues, and (iii) revealed various domains having putative important roles that should be experimentally studied in the future (Figure 2).

### Mutation analyses

#### Genotypes

**Figure 3:**
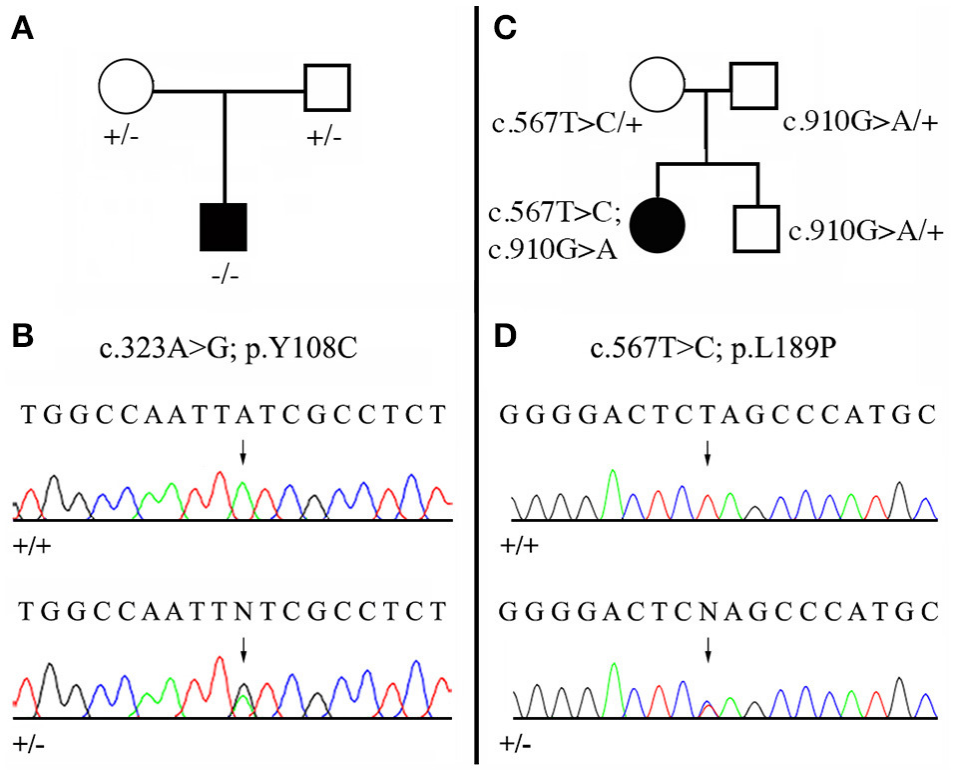
Mutational analysis of the two new *MMP20* mutations. **(A,C)** Pedigree of the AI kindred; **(B,D)** DNA sequencing chromatograms of control (+/+) and of heterozygous (+/−) mutations. Arrows point to the mutation sites. See Figure 2, Supplementary Figure 1, Supplementary Table 2 for the validation of these mutations by means of evolutionary analyses. **(A,B)** Patient 1 with the homozygous mutation (c.323 A>G; p.Y108C). **(C,D)** Patient 2 with the compound heterozygous mutation (c.567 T>C; p.L189P / c.910 G>A; p.A304T). The second mutation was already reported as homozygous *MMP20* mutation (Lee et al., 2010).

Among our patients displaying non-syndromic AI, mutations in the *MMP20* coding gene were diagnosed in six, unrelated families. Clinical diagnoses were confirmed through sequencing as described below. Sequencing DNA of patients 1 and 2 revealed new mutations in the *MMP20* sequence, which are validated as being responsible for the AI phenotype by means of evolutionary analysis. The pedigrees and DNA sequencing chromatograms are presented in Figure 3. Moreover, patient 4 displayed two, already reported mutations, but in a new, compound heterozygous context. In patient 3, the compound heterozygous mutations were already described by Prasad et al. (2016a) but not illustrated (see below). Eventually, patients 5 and 6 possessed an already described homozygous mutation. In addition, in these six families we identified several SNPs that change the amino acid but these mutations are not validated by our evolutionary analysis as they occurred in variable positions.

##### Patient 1: homozygous mutation c.323 A>G

We identified a missense, homozygous mutation in exon 2 of *MMP20* of this male proband. The mutation was not previously reported and is referred to as g.8,470 A>G, c.323 A>G, p.Y108C. Both unaffected parents were heterozygous (Figures 3A,B). The mutation occurred at a tyrosine residue of the catalytic domain, a position that is unchanged in the 75 MMP20 mammalian sequences studied and surrounded by a number of conserved residues of this domain (Figure 2, Supplementary Figures 1, 2). This finding indicates a putative important function for this amino acid and validates this homozygous mutation as being responsible for the AI phenotype.

A second missense, homozygous mutation was identified in exon 1, p.K18T. This frequent mutation in the human population occurred in a variable region of MMP20 and is not responsible for the enamel disorder.

##### Patient 2: compound heterozygous mutation c.567 T>C and c.910 G>A

In this female proband, we identified two missense, heterozygous mutations that were validated as responsible for AI phenotype in a compound genotype. The mutation on allele 1 is located in exon 6 and referred to as g.18,755 G>A, c.910 G>A, p.A304T. This *MMP20* mutation was already reported in the literature as responsible for the AI phenotype in a patient homozygous for the mutation (Lee et al., 2010). The patient 2 was heterozygous for this mutation, as well as his unaffected father and his brother (Figure 3C). The second mutation, on allele 2 was not previously reported and is located in exon 4 and referred to as g.15,345 T>C, c.567 T>C, p.L189P. The patient 2 was heterozygous for this mutation, as well as her unaffected mother (Figures 3C,D). The substitution of a leucine with a proline occurred at a conserved position, unchanged in mammals, and surrounded by numerous conserved residues. The substitution of this putative, functionally, or structurally important amino acid validates the mutation as involved in the AI phenotype.

In addition, the MMP20 sequence of patient 2 displayed four other missense mutations of various functional weight when considering our evolutionary analysis (Figure 2, Supplementary Figures 1, 2): (i) An uncommon missense mutation, on allele 2, was found in exon 3 and referred to as g.13,560 A>C, c.505 A>C, p.I169L. The mother is heterozygous for this mutation. The substitution of the isoleucine with a leucine occurred at a conservative position, at which isoleucine is substituted with the only valine in a few mammalian species but not with leucine (Figure 2, Supplementary Figures 1, 2). These amino acids have, however, similar properties. Also, this mutation occurred in a position surrounded by many conserved residues. The missense mutation p.I169L is present in 3% of the human population (the 1,000 genomes project). The involvement of this missense mutation in the AI phenotype is therefore quite doubtful; (ii) three, common, missense mutations were identified in exon 1, p.K18T (as in patient 1) and p.P31L, and in exon 6, p.T281N, also located in a variable region. These mutations are frequent in the human population occurred in variable regions of MMP20 and are not responsible for the enamel disorder.

The compound heterozygous mutation c.567 T>C (p.L189P) and c.910 G>A (p.A304T) is validated by our evolutionary analysis as the two mutations are located in conserved domains of MMP20, indicating putative important functions for these two amino acids.

##### Patient 3: compound heterozygous mutation c.126+6 t>g and c.954-2 a>t

The compound heterozygous mutation of *MMP20* in intronic splicing sites identified in this male proband was recently reported in the literature (Prasad et al., 2016a) but was not detailed and not documented with pictures (see below).

The mutation on allele 1 is located at the splicing acceptor site of intron 6 (3′ splice site) and referred to as g.30574 a>t, c.954-2 a>t, p.I319fs338X. This MMP20 mutation disturbing the splice site consensus sequence for introns was already reported in the literature as responsible for the AI phenotype, but in a homozygous context (Kim et al., 2005). Patient 3 was heterozygous for the mutation, as well as his unaffected mother. The mutation on allele 2 is located at the splicing donor site of intron 1 (5′ splice site) and referred to as g.145 t>g, c.126+6 t>g. The male proband was heterozygous for the mutation as well as his unaffected father. This substitution did not occur during 180 million years of mammalian evolution (Supplementary Table 2) and this position is therefore considered of importance for correct intron 1 splicing. In addition, (i) the position +6 belongs to the splice site consensus sequence of the 5′ splice site for introns (A/CAG | gta/gag**t**), (ii) Netgen2 server predicted that the splice donor site in intron 1 does not exist in the mutant sequence and MaxEntScan analysis showed a reduced score of the splicing signal in the mutant compared to the wild sequence (Supplementary Data 2), and (iii) this mutation is not present in the human population (1,000 genomes project).

In addition, three missense mutations, common in the human population (p.K18T, V275A, and p.N281T) were also identified in this patient but are not responsible for the enamel disorder.

##### Patient 4: compound heterozygous mutation c.389 C>T and c.954-2 a>t<

We identified two already reported mutations in this male proband. These mutations were, however, described as homozygous *MMP20* mutations in two, unrelated patients, and are reported here to occur as a compound heterozygous mutation in the same patient. The first mutation found on allele 1 and located in exon 3 is referred to as: g.13444 C>T, c.389 C>T, p.T130I (Gasse et al., 2013). Patient 4 was heterozygous for the mutation as well as his unaffected father. The second mutation on allele 2 occurred at the splicing acceptor site of intron 6 and was referred to as g.30574 a>t, c.954-2 a>t, p.I319fs338X (Kim et al., 2005). The male proband was heterozygous for the mutation as well as his unaffecte mother.

##### Patients 5 and 6: homozygous mutation c.954-2 a>t

Patient 5: The homozygous mutation at the splicing acceptor site of intron 6 identified also in patients 3 and 4 was identified in two sisters of a family, in which the mother, the father, two sisters and a brother were unaffected as heterozygous for thed mutation.

Patient 6: The same mutation was also found in two sisters of this family.

All patients sharing the c.954-2 a>t mutation were from unrelated families, a finding that could indicate a high frequency of this mutation in the human population in an heterozygous context.

#### Clinical phenotypes

**Figure 4:**
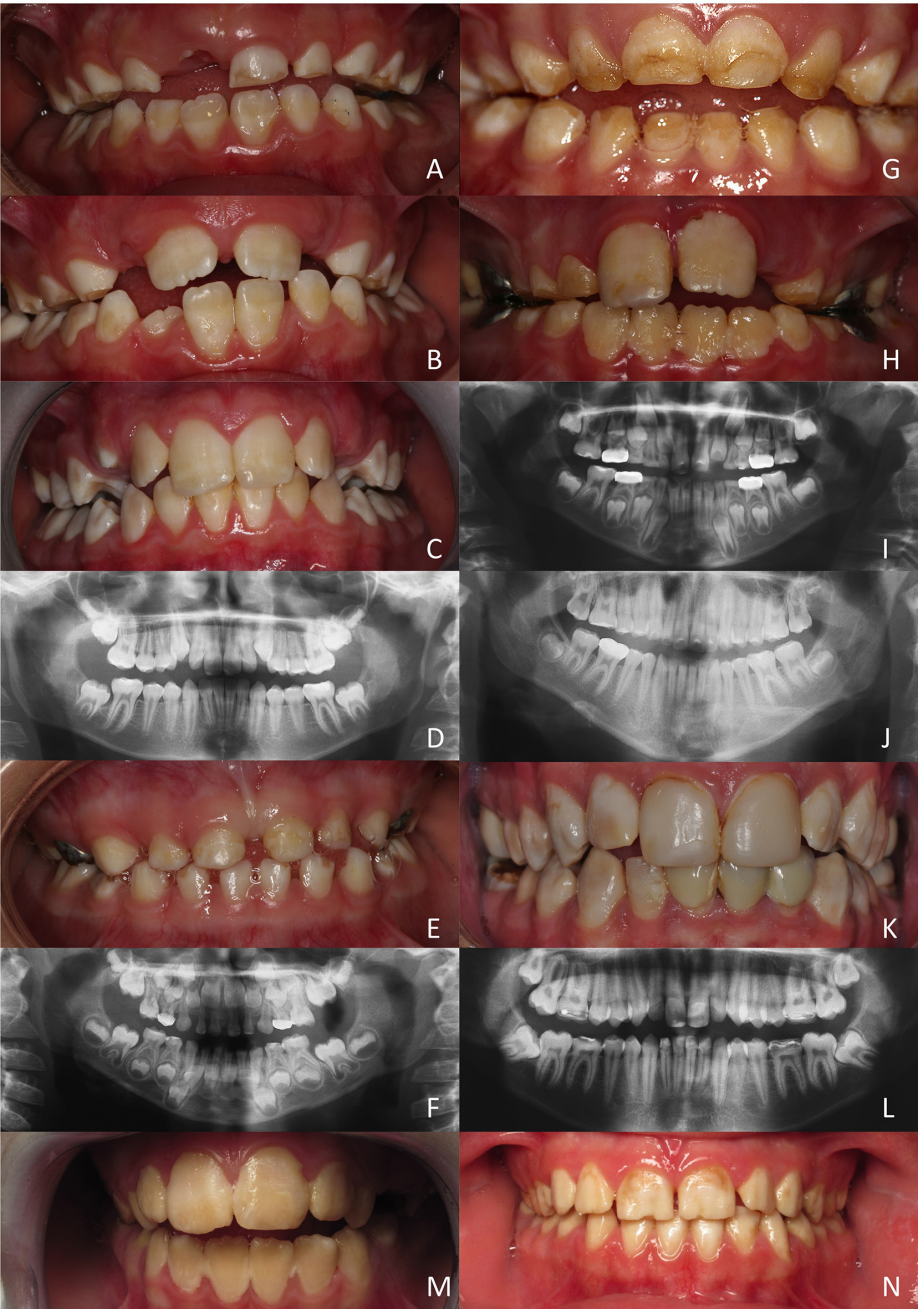
Hypomature amelogenesis imperfecta encountered in patients with diverse *MMP20* mutations. **(A–D):** Patient 1 (c. 323 A>G). Intraoral clinical views and panoramic radiograph of primary, mixed (**A**: 6 years old; **B**: 7 years old) and permanent (**C,D**: 11 year old) dentitions. Note the limited contrast between enamel and dentine on X-rays. **(E,F)**: Patient 2 (c.567 T>C + c.910 G>A). Primary dentition of a 5 year old girl. No enamel or very thin enamel was visible on X-rays. **(G–J)**: Patient 3 (c.126+6 t>g + c.954-2 a>t). A young boy at 5 (**G,I**: primary teeth) and then at 8 years (**H,J**: permanent teeth). Limited radio-opaque enamel, if none, was seen on X-rays. **(K, L)**: Patient 4 (c.389 C>T + c.954-2 a>t). Permanent teeth of a 20-year old man. Hypomature enamel is clearly visible on X-rays. **(M,N)**: Patient 5 and patient 6 (c.954-2 a>t). Two girls displaying the same mutation leading to similar phenotypes with hypomature amelogenesis imperfecta.

Here below is a brief description of the features for the six patients displaying non-syndromic hypomature AI and diagnosed as possessing mutations in the *MMP20* coding gene (Figure 4).

##### Patient 1 (Figures 4A–D)

All teeth of this young boy were affected and parents reported damaged teeth since eruption. Enamel was chalky white and opaque. In primary teeth, enamel was either hypoplastic and/or was prematurely shed and worn through mastication and occlusal forces. The panoramic radiograph revealed the poor contrast of enamel compared to dentine, confirming the under-mineralization of enamel.

##### Patient 2 (Figures 4E,F)

This 5 years old girl was in her primary dentition. The parents reported that primary tooth eruption was delayed as no teeth were present at 1 year. As soon as teeth erupted they showed more opaque enamel and it crumbled. Teeth were small microdont and numerous diastema separated them. Enamel was white, orangy and wore off. On panoramic radiograph, no or very thin enamel was visible on primary teeth. In non-erupted permanent teeth, enamel seemed thicker and more mineralized with a stronger differential contrast with dentine, at least on the first permanent molar germs.

##### Patient 3 (Figures 4G–J)

In this 5 years old boy, the enamel of primary teeth was more opaque and was prone to disintegration, leaving areas of dentine apparent. In permanent teeth, enamel was also colored and opaque. Erupting teeth were sensitive and the patient experienced difficulties to brush teeth. Dental plaque and gingivitis were clearly visible. On panoramic radiographs limited radiopaque enamel if none was visible, and no contrast existed between enamel and dentine. This patient displayed a severe phenotype.

##### Patient 4 (Figures 4K,L)

In this 20 year old man, all permanent teeth demonstrated colored, opaque white brownish teeth with hypomature enamel. On the panoramic radiograph enamel is thin and the contrast with dentine is hardly visible.

##### Patient 5 and patient 6 (Figures 4M,N)

These two girls from unrelated families shared the same mutation and displayed similar phenotypes with colored hypomature amelogenesis imperfecta. The overall tooth contour was respected and enamel chipping was visible at the incisal edge.

#### Phenotype comparison

Patient 1 (p.Y108C) was the least affected patient, while the most severe case was patient 3 displaying two mutations in splicing sites, leading to porous enamel and very sensitive teeth. Patient 2′s phenotype was slightly different as it presented additional quantitative defect associated to hypoplastic AI (smaller teeth). The enamel of patients 4, 5, and 6 showed similar mottled appearance of the enamel with more or less irregular staining. In patients 5 and 6 enamel looked rather opaque and uniformly colored.

The comparison of our case series with patients' phenotypes published in the literature led us to the following observations: Patient 1 phenotype was close to the one described for patient 1 by Gasse et al. (2013), characterized by a stronger contrast between enamel and dentine on X-rays. The enamel phenotype of patient 4 was similar to the affected individual described by Kim et al. (2005, Figure 1), to patient 2 described by Gasse et al. (2013), and to our patients 5 and 6. However, an anterior openbite was not present. Taurodontism was seen in molars especially in the upper permanent molars.

### MMP20 mutations known to date and validation using evolutionary analysis

**Figure 5:**
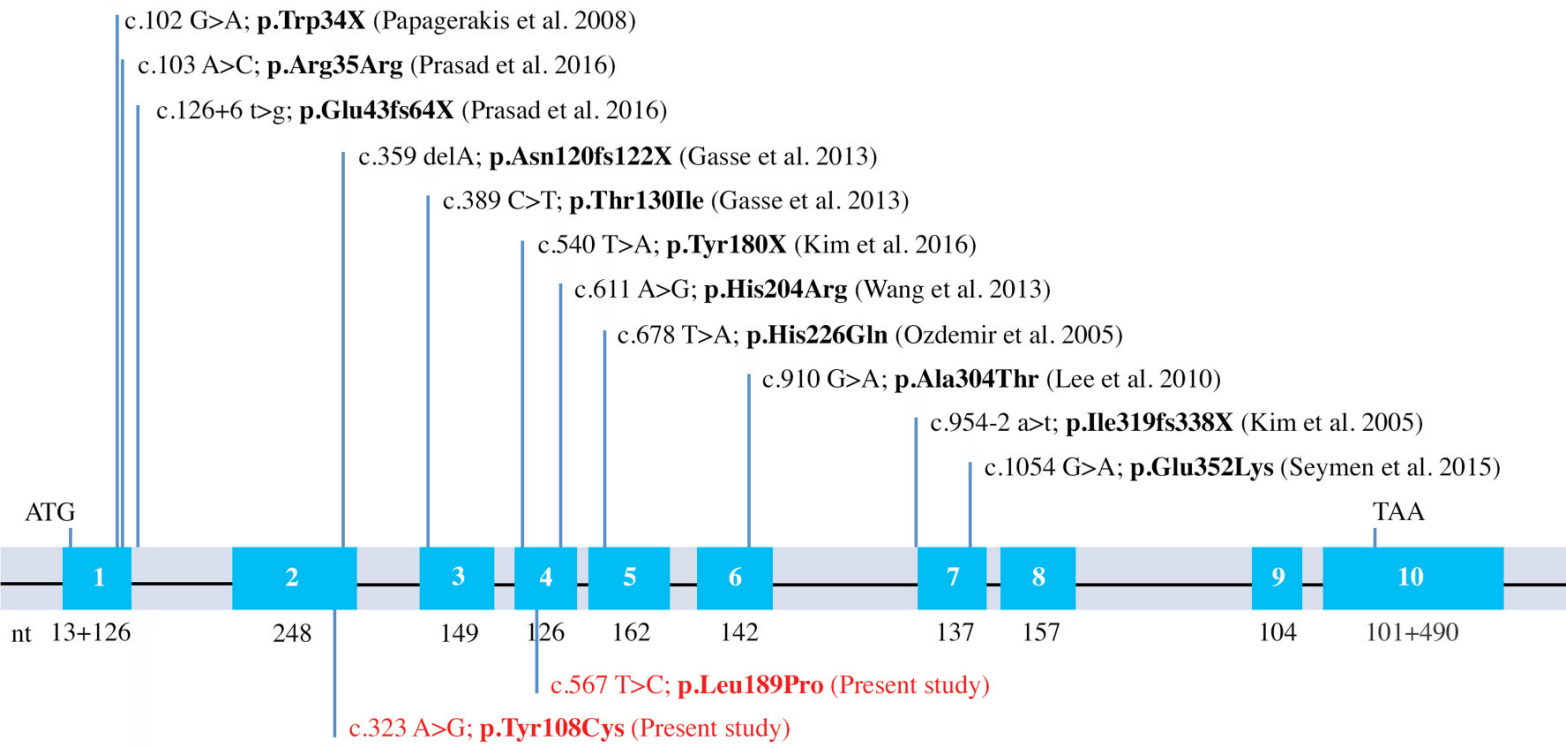
*MMP20* mutations known to date. Schematical representation of the human *MMP20* gene structure with indication of the previously reported mutations and the two new mutations identified in this study (in red).

When including the two new mutations reported in the present study, there are now 13 different *MMP20* mutations leading to AI reported in the literature. Eight of them are simply missense mutations leading to the only substitution of an amino acid (Figures 2, 5). This finding demonstrates the crucial importance of these residues for the correct function of this protease. With the exception of p.Arg35Arg, which concerns the substitution of a nucleotide only (see discussion in Prasad et al., 2016a), the seven other residue substitutions in MMP20 are validated by our evolutionary analysis as occurring either on a conserved (unchanged residue: 5 cases) or on a conservative (2 cases) position.

To date, 18 other genes have been shown causing non-syndromic AI: *AMELX* (16 different mutations; Kim et al., 2017), *FAM83H* (14, Pourhashemi et al., 2014), *WDR72* (10, Hentschel et al., 2016), *ENAM* (4; Pavlic et al., 2007; Seymen et al., 2014), *ITGB6/4* (4; Poulter et al., 2014; Wang et al., 2014b), *SLC24A4* (3; Wang et al., 2014b), *LAMA3* (3, Gostyńska et al., 2016), *GPR68* (3, Parry et al., 2016b), LTBP3 (3; Huckert et al., 2015), *AMBN* (2; Prasad et al., 2016a), *KLK4* (2; Lu et al., 2008; Wang et al., 2013), *DLX3* (2; Kim et al., 2016b), *STIM1* (2; Wang et al., 2014b; Parry et al., 2016a), *COL17A1* (1; Prasad et al., 2016b), *C4orf26* (1; Prasad et al., 2016b), *LAMB3* (1; Kim et al., 2016c), *ACPT* (1, Seymen et al., 2016), and *AMTN* (1, Smith et al., 2016). A total of 13 different mutations on the gene sequence causing AI places MMP20 among the top three sensitive proteins involved in non-syndromic AI when mutated.

Providing a clear genetic diagnosis linking genotype and phenotype on the basis of a missense variant can be challenging. A mutation present in a region that is highly conserved in evolution suggests that the amino acid is functionally important. The validated evolutionary analysis is crucial to address these important conserved positions and to facilitate variant analysis leading to disease diagnosis.

## Author contributions

BG, MP, SD, AB, and JS have substantially contributed to the conception, design of the work and interpretation of data for the work; MH, MK, AG, SL, IB, and MM have substantially contributed to the acquisition or analysis of data. BG, AB, and JS have drafted the work and MP, SD, MH, MK, AG, SL, IB, and MM revised it critically for important intellectual content. All authors finally approved the version to be published; they all agree to be accountable for all aspects of the work and ensure that questions related to the accuracy or integrity of any part of the work are appropriately investigated and resolved.

### Conflict of interest statement

The authors declare that the research was conducted in the absence of any commercial or financial relationships that could be construed as a potential conflict of interest. The reviewer EB and handling Editor declared their shared affiliation, and the handling Editor states that the process nevertheless met the standards of a fair and objective review.
