# Supplementary material for: Evolutionary Analysis Predicts Sensitive Positions of MMP20 and Validates Newly- and Previously-Identified MMP20 Mutations Causing Amelogenesis Imperfecta
Source: Front Physiol. 2017 Jun 14;8:398. doi: 10.3389/fphys.2017.00398 (PMC5469888; doi:10.3389/fphys.2017.00398)
Supplement: Supplementary file 1 [file Table1.PDF]

Supplementary Table 1. Scientific names, preferred common names, families, orders and references in GenBank for the 75 mammalian species, from which *MMP20* sequences were used in our study. Published mRNA sequences in bold. Amino acid sequences are available in Supplementary Data 1.

Genus and species	Common name	Family	Order	Source
Homo sapiens	Human	Hominidae	Primates	NM_004771.3
Pan troglodytes	Chimpanzee	Hominidae	Primates	XM_001153208.3
Gorilla gorilla	Gorilla	Hominidae	Primates	XM_004052031.1
Pongo pygmaeus	Orangutan	Hominidae	Primates	XM_002822398.2
Nomascus leucogenys	Gibbon	Hylobatidae	Primates	ENSNLET00000009150
Papio anubis	Baboon	Cercopithecidae	Primates	XM_003910598.2
Mandrillus leucophaeus	Drill	Cercopithecidae	Primates	XM_011984398.1
Macaca mulatta	Macaque	Cercopithecidae	Primates	NM_001194077.1
Chlorocebus sabaeus	Green monkey	Cercopithecidae	Primates	XM_008020664.1
Rhinopithecus roxellana	Snub-nosed monkey	Cercopithecidae	Primates	XM_010379059.1
Colobus angolensis	Angola colobus	Cercopithecidae	Primates	XM_011926628.1
Callithrix jacchus	Marmoset	Cebidae	Primates	XM_002754349.2
Saimiri boliviensis	Squirrel monkey	Cebidae	Primates	XM_003923773.1
Aotus nancymaae	Night monkey	Aotidae	Primates	XM_012455126.1
Tarsius syrichta	Tarsier	Tarsiidae	Primates	XM_008055968.1
Otolemur garnettii	Bushbaby	Galagidae	Primates	XM_003780797.1
Daubentonia madagascariensis	Aye-Aye	Daubentoniidae	Primates	DauMad_1.0
Microcebus murinus	Mouse lemur	Cheirogaleidae	Primates	ENSMICT00000002937
Galeopterus variegatus	Sunda flying lemur	Cynocephalidae	Dermoptera	XM_008579626.1
Tupaia chinensis	Tree shrew	Tupaiidae	Scandentia	XM_006150092.1
Mus musculus	Mouse	Muridae	Rodentia	NM_013903.2
Rattus norvegicus	Rat	Muridae	Rodentia	NM_001106800.1
Cricetulus griseus	Chinese hamster	Cricetidae	Rodentia	XM_003496135.2
Mesocricetus aureus	Golden hamster	Cricetidae	Rodentia	XM_005329098.1
Microtus ochrogaster	Prairie vole	Cricetidae	Rodentia	XM_005346781.1
Jaculus jaculus	Jerboa	Dipodidae	Rodentia	XM_004661906.1
Dipodomys ordii	Kangaroo rat	Heteromyidae	Rodentia	ENSDORT00000015101
Cavia porcellus	Guinea pig	Caviidae	Rodentia	XM_003472776.1
Octodon degus	Degu	Octodontidae	Rodentia	XM_004626149.1
Chinchilla lanigera	Chinchilla	Chinchillidae	Rodentia	XM_005377981.1
Fukomys damarensis	Damara mole rat	Bathyergidae	Rodentia	XM_010642049.1
Heterocephalus glaber	Naked mole rat	Bathyergidae	Rodentia	XM_004870794.1
Nannospalax galili	Mountain blind mole rat	Spalacidae	Rodentia	XM_008829897.1
Ictidomys tridecemlineatus	Squirrel	Sciuridae	Rodentia	XM_005329098.1
Oryctolagus cuniculus	Rabbit	Leporidae	Lagomorpha	XM_002708568.1
Ochotona princeps	Pika	Ochotonidae	Lagomorpha	XM_004585200.1
Bos taurus	Cow	Bovidae	Cetartiodactyla	NM_174391.2
Capra hircus	Goat	Bovidae	Cetartiodactyla	XM_005689365.1
Ovis aries	Sheep	Bovidae	Cetartiodactyla	XM_004015973.1
Pantholops hodgsonii	Tibetan antelope	Bovidae	Cetartiodactyla	XM_005970051.1
Tursiops truncatus	Dolphin	Delphinidae	Cetartiodactyla	ENSTTRT00000014736
Orcinus orca	Killer whale	Delphinidae	Cetartiodactyla	XM_004282198.1
Lipotes vexillifer	Yangtze river dolphin	Lipotidae	Cetartiodactyla	XM_007448555.1
Physeter catodon	Sperm whale	Physeteridae	Cetartiodactyla	XM_007115662.1
Vicugna pacos	Alpaca	Camelidae	Cetartiodactyla	XM_006206699.1
Camelus ferus	Camel	Camelidae	Cetartiodactyla	XM_006186197.1
Sus scrofa	Pig	Suidae	Cetartiodactyla	ENSSSCT00000016345
Equus caballus	Horse	Equidae	Perissodactyla	XM_001500052.2
Ceratotherium simum	Rhinoceros	Rhinocerotidae	Perissodactyla	XM_004427400.1
Canis familiaris	Dog	Canidae	Carnivora	XM_849546.1
Ailuropoda melanoleuca	Giant panda	Ursidae	Carnivora	ENSAMET00000011054
Ursus maritimus	Polar bear	Ursidae	Carnivora	XM_008690151.1
Leptonychotes weddellii	Weddell seal	Phocidae	Carnivora	LepWed1.0
Odobenus rosmarus	Walrus	Odobenidae	Carnivora	XM_004415992.1
Mustela putorius furo	Ferret	Mustelidae	Carnivora	XM_004764459.1
Felis catus	Cat	Felidae	Carnivora	XM_003992304.1
Erinaceus europaeus	Hedgehog	Erinaceidae	Erinaceomorpha	XM_007520728.1
Sorex araneus	Shrew	Soricidae	Soricomorpha	XM_004604811.1
Condylura cristata	Star-nosed mole	Talpidae	Soricomorpha	XM_004689048.1
Myotis lucifugus	Microbat	Vespertilionidae	Chiroptera	XM_006094667.1
Pteropus vampyrus	Large flying fox	Pteropodidae	Chiroptera	ENSPVAT00000015451
Eidolon helvum	Straw-colored fruit bat	Pteropodidae	Chiroptera	AWHC01
Eptesicus fuscus	Big brown bat	Vespertilionidae	Chiroptera	XM_008149202.1
Rhinolophus hipposideros	Horseshoe bat	Rhinolophidae	Chiroptera	AWHA01
Pteronotus parnellii	Common mustached bat	Mormoopidae	Chiroptera	AWGZ01
Megaderma lyra	Greater false vampire bat	Megadermatidae	Chiroptera	AWHB01
Loxodonta africana	Elephant	Elephantidae	Proboscidea	ENSLAFT00000010998
Procavia capensis	Hyrax	Procaviidae	Hyracoidea	ENSPCAT00000004300
Elephantulus edwardii	Elephant shrew	Macroscelididae	Macroscelidea	XM_006887307.1
Echinops telfairi	Tenrec	Tenrecidae	Afrosoricida	XM_004709009.1
Chrysochloris asiatica	Golden mole	Chrysochloridae	Afrosoricida	XM_006833772.1
Trichechus manatus	Manatee	Trichechidae	Sirenia	XM_004385860.1
Sarcophilus harrisii	Tasmanian devil	Dasyuridae	Dasyuromorphia	XM_003764279.1
Monodelphis domestica	Opossum	Didelphidae	Didelphimorphia	XM_007494975.1
Macropus eugenii	Wallaby	Macropodidae	Diprotodontia	ENSMEUT00000006419
