# Supplementary material for: Evolutionary Analysis Predicts Sensitive Positions of MMP20 and Validates Newly- and Previously-Identified MMP20 Mutations Causing Amelogenesis Imperfecta
Source: Front Physiol. 2017 Jun 14;8:398. doi: 10.3389/fphys.2017.00398 (PMC5469888; doi:10.3389/fphys.2017.00398)
Supplement: Supplementary file 2 [file Table2.PDF]

Supplementary Table 2. Alignment of the untranslated DNA sequences (21 nucleotides) surrounding the coding sequences of *MMP20* exons in 12 representatives of the main mammalian lineages. Human sequence is used as a reference. Exons are represented with the three first and last letters. Crucial nucleotides of splice acceptor (AG) and donor (GT) sites are in bold. The letter on grey background is the human mutation c.126+6 t>g. (.) = nucleotide identical to human *MMP20* nucleotide; (-) = indel. See supplementary Table 1 for full names.

**Species**	**5′UTR**	**Exon 1**	**5′ Intron 1 3′**	**Exon 2**	**5′ Intron 2**
Homo	ttaccaagctactgtgagggg	ATG // CAG	**gt**ttgtgaatttttgcccaat // cagcctgcctctccatttc**ag**	GCG // CAG	**gt**aatgagatcaagtctttcc
Tarsius	......c...g.c.c.gc.a.	... // ...	.......c..a.......... // ....g......	... // ...	....
Tupaia	............c......a.	... // ...	...c......c........g. // ....t........	..T // ...	...........g......c..
Mus	.c.t.....aa--.a....a.	... // ...	........c.c.c.....c.c //	..C // ...	..........tgc........
Oryctolagus	............cc.....a.	... // ...	..........c.......... // ..at.ca..........g...	..T // ...	...........g.........
Bos	a.c.accaag.ggagat.aa.	... // ...	...g......c.....a.... // ....g......	... // ...	...........ga........
Equus	......c.....c......a.	... // ...	..........c.....a.... // ......a..............	... // ...	...........tgagtc.ct.
Canis	agca.g..gcg.ccc....a.	... // ...	......a...c...a.a.... // ....g..c...	..A // ...	..........gg.........
Myotis	............ct.....a.	... // ...	..........c.....ag... // .....c........c......	..A // ...	...........tgagtc..t.
Sorex	.....ct..cg.at.....a.	... // ...	..........c...c.a.ccc // ....t.......	..A // ...	..........agtc.t.ccag
Loxodonta	....tc......c.g.aa.a.	... // ...	......a...c.....a.... // ....t.t.t.g......	..A // ...	...........ga.....c..
Sarcophilus	..c.a..t.ag..t.aaa.a.	... // ...	...a...g.ac.c...a..t. // tt.....tt...ttc..gt..	..A // ...	.......a.cag...t...aa
	**Intron 2 3′**	**Exon 3**	**5′ Intron 3 3′**	**Exon 4**	**5′ Intron 4**
Homo	atgttccttccgttttcac**ag**	AAT // GAG	**gt**attgtggtactt---ggat // cctttctccccacttgttt**ag**	ATC // ATG	**gt**atatatgcacaattcacca
Tarsius	g.......c.t..........	... // ...	...c....c-....---.cgg // ....gt............	... // ...	....
Tupaia	.........tta.........	... // ...	...c......g..gcttc... // ....t........	... // ...	....
Mus	c....t....--...gtc...	... // ...	.......ac..t..ctt..g. // .aa.caatatttcc.ac....	... // ...	....
Oryctolagus	....g..c.tt..........	.G. // ...	...c....ccg...ctt.... // .g....ctgt...........	... // ...	....
Bos	g....g....t..a..g....	... // ...	...c......g...cct.... // .a...tg.....ta...c...	... // ...	....
Equus	g.........ta.........	... // ...	...c.....gg...ctt.... // .a.......t.g...ac....	... // ...	....
Canis	..........t......g...	... // ...	...c......g.a.ctt.... // .g.....g...g.c...c...	... // ...	....
Myotis	catg.t.c.t.ta........	... // ...	...c......g...ctt.... // .a...................	... // ...	....
Sorex	g....g....t......ct..	... // ...	...c..........att.a.. // ggca.t.t..a...ct.....	... // ...	....
Loxodonta	g......c.tta.........	... // ...	...c......g...ctt.... // .g...................	... // ...	....
Sarcophilus	..t...tc.ttac..gt....	.G. // ...	...a.a.atcct.ctgctcta // tt...t.ttgatg..tgg...	... // ...	....
	**Intron 4 3′**	**Exon 5**	**5′ Intron 5 3′**	**Exon 6**	**5′ Intron 6**
Homo	tttctctctcatattgtct**ag**	GTT // ACG	**gt**aagattattaccttctttg // caactcattttgaatc-ct**ag**	GAC // CCG	**gt**aagcctcagacacgccacc
Tarsius	............t....t...	... // ...	...t.c.c.cc..ac.tc.ct // ...g..tg...t...t-tc..	... // ...	.......cag...g....g.t
Tupaia	.....t...........t...	... // .T.	.......ctc...ac.tc..t // ...g.tgc........-....	... // ...	.......ca....gt.t....
Mus	.........g-.g....t...	.G. // ...	...c...ctcct.acca.c.c // tcttg..c......c.-....	... // TA.	.....t.ca...tg.c.tcag
Oryctolagus	.........tg.gc...g...	... // .T.	...t...g.c...a..tc..t // ...g..cc...cc...-....	... // ...	........a...t.a.a....
Bos	..c...........ca..c..	... // ...	...ca.ccgc...ac.t...t // .g.g...c........-....	... // ...	........g.c.tgt...c..
Equus	.....t...............	... // .T.	...cat.c.c.ca.aat.c.t // ...g...c........-....	... // ...	.....a.agt.c...caat..
Canis	.....g.g..c.tc..ca...	... // ...	...tag.c.c....c..c..t // ...g...c........-....	... // ...	........ag..tga.g...t
Myotis	............g........	... // ...	...ca..cgc..tgc.tc..t // ...g..gc........-....	... // ...	........a.a.tgt..ttaa
Sorex	.c..at...t..g....t...	.C. // ...	...caga.g....tc.gacct // .tgac.....ctct..-....	... // ...	.....ttcaga...g..tt..
Loxodonta	..........c....a.t...	.C. // .T.	...tag.ccc.g.ac.tc..t // ...g..c.........-....	... // ...	......a.a.acttacaggta
Sarcophilus	.a.tc..tct..g..c.t...	.A. // ...	...ta.accc.gaac..a.ct // actt.at.........t.c..	... // .A.	..g...tc..t.t.tattcaa
	**Intron 6 3′**	**Exon 7**	**5′ Intron 7 3′**	**Exon 8**	**5′ Intron 8**
Homo	tttttttggaatgtcccat**ag**	GAT // AAG	gtaccctacagatccctcaaa // aactttaatttctgttttc**ag**	GTC // CAG	**gt**atggcttttttccttttta
Tarsius	.....c.....ca....c...	... // ...	..............t...... // g....gcg.............	... // ...	...a.......c.......g.
Tupaia	gac.....a..ca...a.c..	... // ...	.......g.t..c...c.... // ....c................	.C. // ...	.....ct...c.c...c.g.t
Mus	..c.....acccac..a....	... // ...	.....-........t.gt.tg // g...c...cc.t.....c...	... // ...	..gc..gc..cact.c.....
Oryctolagus	..c.....a..cac..a.c..	... // ...	......c.............. //	... // ...	...........c...a.c.gg
Bos	.cc....ctgt..c.aa....	... // ...	...ag..ga....t..g.g.. // .g............g..g...	.A. // ...	...........c..t..g.g.
Equus	..c......at..c..a....	... // ...	......c......ttcc.... // ....c........	.C. // ...	.....ct...c.c.t...gat
Canis	c....g....t..c..a.c..	... // ...	.........t......cag.g //	.C. // ...	...........c.......g.
Myotis	......g..gt..c..a....	... // ...	.........g...t....g.. // ....c.......g.t..	.C. // ...	.....ca...cc.........
Sorex	.c.c..g.act..c..a....	... // ...	....ttgg.ca.c.....ctg // ..c.c.gg.....a.......	.G. // ...	...ca..c.cc.ctac.c.cg
Loxodonta	c.c........ca...a....	... // ...	...t.....tatg.ttg...c // ....c.g..............	... // ...	...........c.....cg.c
Sarcophilus	cc.g...t.gg.aa..a....	... // ...	.....ta..ca.at..c.t.c // c.t.c.ttg......gc.t..	... // ...	...cttt.accagtgc..a.t

	Intron 8 3′	Exon 9	5′ Intron 9 3′	Exon 10	3'UTR
Homo	tgaaata-atacttttct**ag**	CTA // ATG	**gt**aagtgtttccactgaaaac // tttcaactttgtctctttt**ag**	GCT // TAA	atagaaaagcctagtcttctc
Tarsius	.a..g..-.cg...c..tc..	... // ...	..g.........tca.g... // ....t......	... // ...	.........ttcctgga.ggg
Tupaia	..-....-......c...c..	T.. // ...	..g.......aa...tt.tgg //	... // ...	g......g.t..t.....t.t
Mus	..--.a.-ta.ta....tc..	... // ...	..ga..t.gc....ca....t // ....a......c...	... // .G.	g.c.tc.g.t..ctc.gcagg
Oryctolagus	.......-......c...c..	... // ...	..g..........cag...a //	... // ...	...a....at..t....atc.
Bos	.......-...t.cc..ac..	T.. // ...	..g..........tg...c.. // .....t...............	... // ...	....c....t........tc.
Equus	...---.-..ga.ac...c..	... // .C.	..ga..........ca..... //	... // ...	.c...g...t........tc.
Canis	t......-......c...c..	... // ...	..g..ca.c.....cc..... // ....c................	... // ...	.c....gg.t..c..g..t..
Myotis	.......-......cc..c..	... // ...	..g...a...t...ca....a //	... // ...	.g.....g.t..g.....tc.
Sorex	.ctgt..-......c...c..	... // ...	..g...a...t...ac.c.ca // ..catcg...t...tg.....	... // ...	.c..g..gctgccaag.ctc.
Loxodonta	.......-.c....c..cc..	... // ...	..g..c....tgtt.tgtttt // .c...................	... // ...	.........t........tc.
Sarcophilus	.......tc.ta...g.....	T.. // ...	..g...acc.tat.aa..ctg // c..tc.a...a..........	.T. // ...	g..ag..cataa...a.gaag
