# Supplementary material for: Evolutionary Analysis Predicts Sensitive Positions of MMP20 and Validates Newly- and Previously-Identified MMP20 Mutations Causing Amelogenesis Imperfecta
Source: Front Physiol. 2017 Jun 14;8:398. doi: 10.3389/fphys.2017.00398 (PMC5469888; doi:10.3389/fphys.2017.00398)
Supplement: Supplementary file 3 [file DataSheet1.PDF]

Supplementary Data 1. Amino acid sequences of the 75 mammalian MMP20 used in this study. (?) = unknown residue; (*) = stop codon. See supplemental file S1 for species names and references.

```
Primates
>Homo
MKVLPASGLAVFLIMALKFSTAAPSLVAASPRTWRNNYRLAQAYLDKYYTNKEGHQIGEMVARGSNSMIRKIKELQAFFGLQVTGKLDQTTMNVIKKPRCGVPDV
ANYRLFPGEPKWKKNTLTYRISKYTPSMSSVEVDKAVEMALQAWSSAVPLSFVRINSGEADIMISFENGDHGDSYPFDGPRGTLAHAFAPGEGLGGDTHFDNAEK
WTMGTNGFNLFTVAAHEFGHALGLAHSTDPSALMYPTYKYKNPYGFHLPKDDVKGIQALYGPRKVFLGKPTLPHAPHHKPSIPDLCDSSSSFDAVTMLGKELLLF
KDRIFWRRQVHLRTGIRPSTITSSFPQLMSNVDAAYEVAERGTAYFFKGPHYWITRGFQMQGPPRTIYDFGFPRHVQQIDAAVYLREPQKTLFFVGDEYYSYDER
KRKMEKDYPKNTEEEFSGVNGQIDAAVELNGYIYFFSGPKTYKYDTEKEDVVSVVKSSSWIGC*
>Pan
MKVLPASGLAVFLIMALKFSTAAPSLVAASPRTWRNNYRLAQAYLDKYYTNKEGHQIGEMVARGSNSMIRKIKELQAFFGLQVTGKLDQTTMNVIKKPRCGVPDV
ANYRLFPGEPKWKKNILTYRISKYTPSMSSVEVDKAVEMALQAWSSAVPLSFVRINSGEADIMISFENGDHGDSYPFDGPRGTLAHAFAPGEGLGGDTHFDNAEK
WTMGTNGFNLFTVAAHEFGHALGLAHSTDPSALMYPTYKYKNPYGFHLPKDDVKGIQALYGPRKAFLGKPTLPHAPHHKPSIPDLCDSSSSFDAVTMLGKELLLF
KDRIFWRRQVHLRTGIRPSTITSSFPQLMSNVDAAYEVAERGTAYFFKGPHYWITRGFQMQGPPRTIYDFGFPRHVQQIDAAVYLREPQKTLFFVGDEYYSYDER
KKKMGKDYPKNTEEEFSGVNGQIDAAVELNGYIYFFSGPKTYKYDTEKEDVVSVVKSSSWIGC*
>Gorilla
MKVLPASGLAVFLIMALKFSTAAPSLVAASPRTWRNNYRLAQAYLDKYYTNKEGHQIGEMVARGSNSMIRKIKELQAFFGLQVTGKLDQTTMNVIKKPRCGVPDV
ANYRLFPGEPKWKKNTLTYRISKYTPSMSSVEVDKAVEMALQAWSSAVPLSFVRINSGEADIMISFENGDHGDSYPFDGPRGTLAHAFAPGEGLGGDTHFDNAEK
WTMGTNGFNLFTVAAHEFGHALGLAHSTDPSALMYPTYKYKNPYRFHLPKDDVKGIQALYGPRKAFLGKPTLPHAPHHKPSIPDLCDSSSSFDAVTMLGKELLLF
KDRIFWRRQVHLRTGIRPSTITSSFPQLMSNVDAAYEVAERGTAYFFKGPHYWITRGFQMQGPPRTIYDFGFPRHVQQIDAAVYLREPQKTLFFVGDEYYSYDER
KRKMEKDYPKNTEEEFSGVNGQIDAAVELNGYIYFFSGPKTYKYDTEKEDVVSVVKSSSWIGC*
>Pongo
MKVLPASGLAVLLIMALKFSTAAPSLVAASPRTWRNNYRLAQAYLDKYYTNKEGHQIGEMVARGGNSMIRKIKELQAFFGLQVTGKLDKTTMNVIKKPRCGVPDV
ANYRLFPGEPKWKKNTLTYRISKYTPSMSSVEVDKAVEMALQAWSSAVPLSFVRINSGEADIMISFENGDHGDSYPFDGPRGTLAHAFAPGEGLGGDTHFDNAEK
WTMGTNGFNLFTVAAHEFGHALGLAHSTDPSALMYPTYKYQNPYGFHLPKDDVKGIQALYGPRKAFLGKPTLPHAPHHKPSTPDLCDSSSSFDAVTMLGKELLLF
KDRIFWRRQVHLRTGIRPSTITSSFPQLMSNVDAAYEVAERGTAYFFKGPHYWITRGFQMQGPPRTIYDFGFPRHVQRIDAAVYLREPQKTLFFVGDEYYSYDER
KRKMEKDYPKNIEEEFSGVNGQIDAAVELNGYIYFFSGPKTYKYDTEKEDVVSVVKSSSWIGC*
>Nomascus
MKVLPASGLAVLLIMALKFSTAAPSLVAASPRTWRNNYRLAQAYLDTYYTNKEGHQTGEMVARGGNSMIRKIKELQAFFGLQVTGKLDQTTMNVIKKPRCGVPDV
ANYRLFPGEPKWKKNTLTYRISKYTPSMSPVEVDKAVEMALQAWSSAAPLSFVRINSGEADIMISFENGDHGDSYPFDGPRGTLAHAFAPGEGLGGDTHFDNAEK
WTMGTNGFNLFTVAAHEFGHALGLAHSTDPSALMYPTYKYKNPYGFHLPKDDVKGIQALYGPRKPFLGKPTVPHAPPYKPSTPDLCDSSSSFDAVTMLGKELLLF
KDRIFWRRQVHLRTGIRPSTITSSFPQLMSNVDAAYEVAERGTAYFFKGPHYWITRGFQMQGPPRTIYDFGFPRHVQRIDAAVYLWEPQKTLFFVGDEYYSYDER
KRKMEKDYPKSTEEEFSGVNGQVDAAVELNGYIYFFSGPKTYKYDTEKEDVVSVVKSSSWIGC*
>Papio
MKVLPASGLAVLLIMALKFSTAAPSLVAASPRTWRNNYRLAQAYLDKYYTNKEGHQIGEMVARGGNSMVRKIKELQAFFGLRVTGKLDQTTMDVIKKPRCGVPDV
ANYRLFPGEPKWKKNILTYRVSKYTTSMSSAEVEKAVQMALQAWSSAVPLSFVRINSGEADIMISFENGDHGDSYPFDGPRGTLAHAFAPGEGLGGDTHFDNAEK
WTMGTNGFNLFTVAAHEFGHALGLAHSTDPSALMYPTYKYQNPYGFHLPKDDVKGIQALYGPRKPFLGKPMVPHAPPHKPSTPDLCDSSSSFDAVTMLGKELLLF
KGRIFWRREVPLRTGIRPSTITSSFPQLMSNVDAAYEVAERGTAYFFKGPHYWITRGFQMQGPPRTIYDFGFPRHVQRIDAAVYLREPQKTLFFVGDEYYSYDER
KRKMEKDYPKNTEEEFSGVNGQIDAAVELNGYIYFFSGPKTYKYDTEKEDVVSVVKSSSWIGC*
>Mandrillus
MKVLPASGLAVLLIMALKFSTAAPSLVAASPRTWRNNYRLAQAYLDKYYTNKEGHQIGEMVARGGNSMVRKIKELQAFFGLRVTGKLDQTTMDVIKKPRCGVPDV
ANYRLFPGEPKWKKNILTYRVSKYTTSMSSAEVEKAVQMALQAWSSAVPLSFVRINSGEADIMISFENGDHGDSYPFDGPRGTLAHAFAPGEGLGGDTHFDNAEK
WTMGTNGFNLFTVAAHEFGHALGLAHSTDPSALMYPTYKYQNPYGFHLPKDDVKGIQALYGPRKPFLGKPMVPHAPPHKPSTPDLCDSSSSFDAVTMLGKELLLF
KGRIFWRRQVHLRTGIRPSTITSSFPQLMSNVDAAYEVAERGTAYFFKGPHYWITRGFQMQGPPRTIYDFGFPRHVQRIDAAVYLREPQKTLFFVGDEYYSYDER
KRKMEKDYPKNTEEEFSGVNGQIDAAVELNGYIYFFSGPKTYKYDTEKEDVVSVVKSSSWIGC*
>Macaca
MKVLPASGLAVLLIMALKFSTAAPSLVAASPRTWRNNYRLAQAYLDKYYTNKEGHQIGEMVARGGNSMVRKIKELQAFFGLRVTGKLDQTTMDVIKKPRCGVPDV
ANYRLFPGEPKWKKNILTYRVSKYTTSMSSAEVEKAVQMALQAWSSAVPLSFVRINSGEADIMISFENGDHGDSYPFDGPRGTLAHAFAPGEGLGGDTHFDNAEK
WTMGTNGFNLFTVAAHEFGHALGLAHSTDPSALMYPTYKYQNPYGFHLPKDDVKGIQALYGPRKPFLGKPMVPHAPHHKPSTPDLCDSSSSFDAVTMLGKELLLF
KGRIFWRRQVHLRTGIRPSTITSSFPQLMSNVDAAYEVAERGTAYFFKGPHYWITRGFQMQGPPRTIYDFGFPRHVQQIDAAVYLREPQKTLFFVGDEYYSYDER
KRKMEKDYPKNTEEEFSGVNGQIDAAVELNGYIYFFSGPKTYKYDTEKEDVVSVVKSSSWIGC*
>Chlorocebus
MKVLPASGLAVLLIMALKFSTAAPSLVAASPRTWRNNYRLAQAYLDKYYTNKEGHQIGEMVARGGNSMVRKIKELQAFFGLRVTGKLDQTTMDVIKKPRCGVPDV
ANYRLFPGEPKWKKNILTYRVSKYTTSMSSAEVEKAVQMALQAWSSAVPLSFVRINSGEADIMISFENGDHGDSYPFDGPRGTLAHAFAPGEGLGGDTHFDNAEK
WTMGTNGFNLFTVAAHEFGHALGLAHSTDPSALMYPTYKYQNPYGFHLPKDDVKGIQALYGPRKPFLGKPMVPHAPAHKPSTPDLCDSSSSFDAVTMLGKELLLF
KGRIFWRRQVHLRTGIRPSTITSSFPQLMSNVDAAYEVAERGIAYFFKGPHYWITRGFQMQGPPRTIYDFGFPRHVQRIDAAVYLREPQKTLFFVGDEYYSYDER
KRKMEKDYPKNTEEEFSGVNGQIDAAVELNGYIYFFSGPKTYKYDTEKEDVVSVVKSSSWIGC*
>Rhinopithecus
MKVLPASGLAVLLIMALKFSTAAPSLVAASPRTWRNNYRLAQAYLDKYYTNKEGHQIGEMVARGGNSMVRKIKELQAFFGLQVTGKLDPTTMDVIKKPRCGVPDV
ANYRLFPGEPKWKKNILTYRISKYTTSMSSAEVEKAVQMALQAWSSAIPLNFVRINSGEADIMISFENGDHGDSYPFDGPRGTLAHAFAPGEGLGGDTHFDNAEK
WTMGKNGFNLFTVAAHEFGHALGLAHSTDPSALMYPTYKYQNPYGFHLPKDDVKGIQALYGPRKPFLGKPMVPHAPPHKPSTPDLCDSSSSFDAVTMLGKELLLF
KGRIFWRRQVHLRTGIRPSTITSSFPQLMSNVDAAYEVAERGTAYFFKGPHYWITRGFQMQGPPRTIYDFGFPRHVQQIDAAVYLREPQKTLFFVGDEYYSYDER
KRKMEKDYPKNIEEEFSGVNGQIDAAVELNGYIYFFSGPKTYKYDTEKEDVVSVVKSSSWIGC*
>Colobus
MKVLPASGLAVLLIMALKFSTAAPSLVAASPRTWRNNYRLAQAYLDKYYTNKEGHQIGEMVARGGNSMVRKIKELQAFFGLQVTGKLDQTTMDVIKKPRCGVPDV
ANYRLFPGEPKWKKNILTYRISKYTTSMSSAEVEKAVQMALQAWSSAIPLNFVRINSGEADIMISFENGDHGDSYPFDGPRGTLAHAFAPGEGLGGDTHFDNAEK
WTMGTNGFNLFTVAAHEFGHALGLAHSTDPSALMYPTYKYQNPYGFHLPKDDVKGIQALYGPRKPFLGKPMVPHAPPHKPSTPDLCDSSSSFDAVTMLGKELLLF
KGRIFWRRQVHLRTGIRPSTITSSFPQLMSNVDAAYEVAERGTAYFFKGPHYWITRGFQMQGPPRTIYDFGFPRHVQQIDAAVYLREPQKTLFFVGDEYYSYDER
KRKMEKDYPKNIEEEFSGVNGQIDAAVELNGYIYFFSGPKTYKYDTEKEDVVSVVKSSSWIGC*
>Callithrix
MKVLPASGLAVLLMMALKFSTAAPSLVAASPRTLRNNYRLAQAYVDKYYTNKEGHQIGEMVSRGGNSLVRKIKELQAFFGLQVTGKLDKTTMDVIKKPRCGVPDV
ANYRLFPGEPKWKKNTLTYRISKYTPSMSPAEVDKAVGMALQAWSGAVPLNFVRINSGEADIMVSFETGDHGDSYPFDGPRGTLAHAFAPGEGLGGDTHFDNAEK
WTMGTNGFNLFTVAAHEFGHALGLAHSTDPSALMYPTYRYQNPYGFHLPKDDVKGIQALYGPRKPFLGKPTVPHAPPHKPSVPDLCDSSSSFDAVTVLGRELLLF
KDRIFWRRQVHLRAGIRPSTITSSFPQLMSNVDAAYEVAERGTAYFFKGPHYWITRGFQMQGPPRSIYDFGFPRHVQRIDAAVYLREPRKTLFFVGDEYYSYDEW
KRKMEKDYPKNTEEEFSGVNGQIDAAVELNGYIYFFSGPKTYKYDTEKEDVVSVVKSSSWIGC*
```

>Saimiri
MKVLPASGLAVLLIMALKFSTAAPSLVAASPRIWRNNYRLAQAYVDKYYTNKEGHQIGEMVSRGGNSMVRKIKELQAFFGLQVTGKLDKTTMDVIKKPRCGVPDV
ANYRLFPGEPKWKKTTLTYRISKYTPSMSPAEVDKAMGMALQAWSSAVPLNFVRINSGEADIMISFETGDHGDSYPFDGPRGTLAHAFAPGEGLGGDTHFDNAEK
WTMGTNGFNLFTVAAHEFGHALGLAHSTDPSALMYPTYKYQNPYGFHLPKDDVKGIQALYGPRKPFLGKPTVPHAPPHKPSTPDLCDSSSSFDAVTMLGKELLLF
KDRIFWRRQVHLRAGIRPSTITSSFPQLMSNVDAAYEVAERGTAYFFKGPHYWITRGFQMQGPPRTIYDFGFPRYVQQIDAAVYLREPQKTLFFVGDEYYSYDER
KRKMEKDYPKNTEEEFSGVNGQIDAAVELNGYIYFFSGPKTYKYDTEKEDVVSVVKSSSWIGC*
>Aotus
MKVLPASGLAVLLIMALKFSTAAPSLVAASPRTWRNNYRLAQAYVDKYYTNKEGHQIGEMVSRGGNSMVRKIKELQAFFGLQVTGKLDKTTMDVIKKPRCGVPDV
ANYRLFPGEPKWKKNTLTYRISKYTPSMSPAEVDKAVGMALQAWSSAVPLNFVRINSGEADIMISFETGDHGDSYPFDGPRGTLAHAFAPGEGLGGDTHFDNAEK
WTMGTNGFNLFTVAAHEFGHALGLAHSTDPSALMYPTYKYQNPYGFHLPKDDVKGIQALYGPRKSFLGKPTLPHAPPHKPSTPDLCDSSSSFDAVTMLGKELLLF
KDRIFWRRQVHLRAGIRPSTITSSFPQLMSNVDAAYQVAERGTAYFFKGPHYWITRGFQMQGPPRTIYDFGFPRHVQRIDAAVYLREPQKTLFFVGDEYYSYDER
KRKMEKDYPKNTEEEFSGVNGQIDAAVELNGYIYFFSGPKTYKYDTEKEDVVSVVKSSSWIGC*
>Tarsius
MKVLPASGLAVLLITALKLSTAAPSLFAATPRTWRNNYHLAQAYLDKYYTRKGGHQVGEMAARGGNSMVRKIKELQAFFGLRVTGKLDPTTMDVIKKPRCGVPDV
ANYRLFPGEPKWKKNTLTYRISKYTSSMSAAEVDKAVEMALQAWSGAVPLSFVRVNSGEADIMISFETGDHGDSYPFDGPRGTLAHAFAPGEGLGGDTHFDNAEK
WTMGMNGFNLFTVAAHEFGHALGLAHSSDPSALMYPTYKYQNPYGFHLPKDDVKGIQALYGPRKPFLGKPTVPHVPPHKPSTPDLCDSSSSFDAVTMLGKELLFF
KDRIFWRRQVHLPAGIRPSTITSSFPQLMSNVDAAYEVAERGTAYFFKGPHYWITRGFRMQGPPRTIYDFGFPRHVQRIDAAVYLREPQKTLFFVGEEYYSYDER
KRKMEKDYPKNTEEEFSGVNGQIDAAVELNGYIYFFSGPKAYKYDTEKEDVVSVVKSSSWIGC*
>Otolemur
MKMLPASGLAVLLITALKLSTAAPSLFTATPRTWRNNYHLAQEYLDKYYTKKGGHQIGEMVARGGNSMVKKIKELQAFFGLQVTGKLDATTMDVIKRPRCGVPDV
ANYRLFPGEPKWKKNTLTYRISKYTPSMSSAEVDTAIEMALRAWSSAVPLNFVRVNTGEADIMISFETGDHGDSYPFDGPRGTLAHAFAPGEGLGGDTHFDNAEK
WTLGMNGFNLFTVAAHEFGHALGLAHSTDPSALMYPTYKYQNPYGFHLPMDDVKGIQALYGPRKPFLGKPTMPHGPPHNPPTPDLCDSSSSFDAVTMLGKELLFF
KDRIFWRRQVHLPTGIRPSTITSSFPQLMSNVDAAYEVAERGTAYFFKGPHYWITRGFQMQGPPRTIYDFGFPRHVQRIDAAVYLREPRKTLFFVGDEYYSYDER
KRKMEKDYPKNTEEEFSGVNGQIDAAVELNGYIYFFSGPKAYKYDMEKEDVVSVVKSSSWVGC*
>Daubentonia
MKMLPASGLAVLLITALKLSTAAPSLFSAAPRTWRNNYHLAQAYLDKYYTKKGEHQIGEVVARGGNSMVKKIKELQAFFGLQVTGKLDRTTMDVIKKPRCGVPDV
ANYRLFPGEPKWKKNTLTYRISKYTPSMSAAEVDKSIEMALRAWSSAVPLNFVRVNAGEADIMISFETGDHGDSYPFDGPRGTLAHAFAPGEGLGGDTHFDNAEK
WTMGMNGFNLFTVAAHEFGHALGLAHSTNPSALMYPTYKYQNPYGFHLPSDDVKGIQALYGPRKPFLGNPSMPHGPPHKPPIPDLCDSSASFDAVTMLGKELLFF
RDRIFWRRQLHLPSGIRPSTITSSFPQLMSNVDAAYEVAERGTAYFFKGPHYWITRGFQMQGPPRTIYDFGFPRHVQQIDAAVYLREPQKTLFFVGDEYYSYDER
KRKMDKDYPKSTEEEFSGVNGQIDAAVELNGYIYFFSGPKAYKYDTEKEDVVSVVKSSSWIGC*
>Microcebus
MKVLPASGLAVLLITALKLSTAAPSLFSATPRTWRNNYHLAQAYLDKYYTKKGGHQIAEMAARGGNSMVKKIKELQAFFGLQVTGKLDPTTMDVIKRPRCGVPDV
ANYRLFPGEPKWKKNTLTYRISKYTPSMSAAEVDKAIDMALRAWSSAVPLNFVRVNAGEADIMISFETGDHGDSYPFDGPRGTLAHAFAPGEGLGGDTHFDNAEK
WTMGMNGFNLFTVAAHEFGHALGLAHSTDPSALMYPTYKYQNPYGFRLPSDDVKGIQALYGPRKPYLGNPAMPHGPPHKPPTPDLCDSSASFDAVTMLGKELLFF
KDRIFWRRQVHLPSGIRPSTITSSFPQLMSNVDAAYEVAERGTAYFFKGPHYWITRGFQMQGPPRTIYDFGFPRHVQRIDAAVYLREPQKTLFFVGDEYYSYDER
KRRMEKDYPKNTEEEFSGVSGQIDAAVELNGYIYFFSGPKAYKYDTEKEDVVSVVKSSSWIGC*

Dermoptera
>Galeopterus
MRVLPASGIAVLLVTALKFSSAAPSLFAATPRTLRNNYHLAQAYLDKYYTKKGGHQIGEMVAKGGSSMVRKIKELQAFFGLQVTGKLDQTTMDVMKRPRCGVPDV
ANYRLFPGEPKWKKNTLTYRISKYTPSMSAVEVDKAVEMALQAWSGAVPLNFVRVLAGEADIMISFETGDHGDSYPFDGPRGTLAHAFAPGEGLGGDTHFDNAEK
WTMGKNGFNLFTVAAHEFGHALGLAHSTDPSALMYPTYKYQDPYDFHLPKDDVKGIQALYGPRKTFLGKPSVPLGPPHRPSIPDLCDSSSYFDAVTMLGKELLFF
RDRIFWRRQVHLMAGIRPSTITSSFPQLMSNVDAAYEVAERGTAYFFKGPHYWITRGFQMQGPARTIYDFGFPRHVQRIDAAVYLKEPQKTLFFVGDEYYSYDER
KRRMEKDYPKSTEEEFSGVNGQIDAAVELNGYIYFFSGPKTYKYDTEKEDVVSVVKSSSWIGC*

Scandentia
>Tupaia
MKVLPASGLAVLLITALKFSSAVPSLFAGTPRSLRNDYHLAQAYLDKYYTRKGGHPIGEMVARGGNSMVKKIKELQAFFGLQVTGKLDQTTMDVIKRPRCGVPDV
ANYRLFPGEPKWKKNTLTYRISKYTPSMSSADVDKAVEMALQAWSSAVPLSFVRVNSGEADIMISFETGDHGDSYPFDGPRGTLAHAFAPGEGLGGDTHFDNAEK
WTMGMNGFNLFTVAAHEFGHALGLAHSTDPTALMYPTYKYQNPYGFHLPKDDVKGIQALYGPRKTFLGKPTVPHAPPHKPSIPDLCDSSSSFDAVTMLGKELLFF
KDRIFWRRQVHLTAGIRPSTITSSFPQLMSNVDAAYEVAERGIAYFFKGPHYWITRGFQMQGPPRTIYDFGFPRHVQRIDAAVYLREPQKTLFFVGDEYYSYDER
KRKMEKDYPKNTEEEFSGVNGQIDAAVELNGYIYFFSGPKAYKYDTEKEDVVSVLKSSSWVGC*

Rodentia
>Mus
MKVLPASGLAVLVTALKFATADPNLLAATPRTFRSNYHLAQAYLDKYYTKKGGPQAGEMVARESNPMIRRIKELQIFFGLKVTGKLDQNTMNVIKKPRCGVPDVA
NYRLFPGEPKWKKNILTYRISKYTPSMSPTEVDKAIQMALHAWSTAVPLNFVRINSGEADIMISFETGDHGDSYPFDGPRGTLAHAFAPGEGLGGDTHFDNAEKW
TMGTNGFNLFTVAAHEFGHALGLGHSTDPSALMYPTYKYQNPYRFHLPKDDVKGIQALYGPRKIFPGKPTMPHIPPHKPSIPDLCDSSSSFDAVTMLGKELLFFK
DRIFWRRQVHLPTGIRPSTITSSFPQLMSNVDAAYEVAERGIAFFFKGPHYWVTRGFHMQGPPRTIYDFGFPRHVQRIDAAVYLKEPQKTLFFVGEEYYSYDERK
KKMEKDYPKNTEEEFSGVSGHIDAAVELNGYIYFFSGRKTFKYDTEKEDVVSVVKSSSWIGC*
>Rattus
MKVLPASGLAVLVTALKFATAAPALLAATPRTFRSNYHLAQAYLDKYYTKKGGPQAGEMVARESNPTVRKIKELQTFFGLRVTGKLDQNTMDVIKRPRCGVPDVA
NYRLFPGEPKWKKNILTYRVSKYTPSMSPTEVDKAVHMALHAWSTAVPLIFVRINSGEADIMISFETGDHGDSYPFDGPRGTLAHAFAPGEGLGGDTHFDNAEKW
TMGTNGFNLFTVAAHEFGHALGLGHSTDPSALMYPTYKYQNPYRFHLPKDDVKGIQALYGPRKTFPGKPTMPHIPPHKPSIPDLCDSSSSFDAVTMLGKELLFFK
DRIFWRRRVHLPAGIRPSTITSSFPQLMSNVDAAYEVAERGIAFFFKGPHYWVTRGFQMQGPPRTIYDFGFPRHVQRIDAAVYLKEPQKTLFFVGEEYYSYDERK
KKMEKDYPKSTEEEFSGVSGHIDAAVELNGYIYFFSGPKTFKYDTEKEDVVSVVKSSSWVGC*
>Cricetulus
MKGLPASGLAVLVTALKFATAAPTLFAAAPRTFRRNYHIAQEYLDKYYSQKGGLQAGEMVARESNPMIRKIKELQSFFGLQVTGKLDQNTMNVIKRPRCGVPDVA
NYRLFPGEPKWKKNILTYRVSKYTSSMSPAEVDKAVEMALRAWSTAVPLNFVRINSGEADIMISFETGDHGDSYPFDGPRGTLAHAFAPGEGLGGDTHFDNAEKW
TMGMNGFNLFTVAAHEFGHALGLGHSTDPSALMYPTYKYQNPSRFHLPKDDVKGIQALYGPRKTFPGKPTVPHIPPHKPSFPDLCDASSSFDAVTMLGKELLLFK
DRIFWRRQVHLAAGIRPNTITSSFPQLMSNVDAAYEVPERGIAFFFKGPHYWITRGFQMQGPPRTIYDFGFPRHVQRIDAAVYLKKPQKTLFFVGEEYYSYDERK
KKMEKDYPKTTEEEFSGVSGQVDAAVELNGYIYFFSGPKTFKYDTEKEDVVSVVKSSSWVGC*
>Mesocricetus
MKVLPASGLAVLVAALKFATAAPTLFAAAPRTSRSSYHIAQEYLDKYYSQKGGLQAGEMVAREDNPMIRKIKELQSFFGLQVTGKLDQNTMNVIKRPRCGVPDVA
NYRLFPGEPKWKKNILTYRVSKYTPSMSPAEVDKAVEMALRAWSTAVPLNFVRINSGEADIMISFETGDHGDSYPFDGPRGTLAHAFAPGEGLGGDTHFDNAEKW
TMGMNGFNLFTVAAHEFGHALGLGHSTDPSALMYPTYKYQNPYRFHLPKDDVKGIQALYGPRKTFPGKPTVPHIPPHKPSFPDLCDTSSSFDAVTMLGKELLLFK
GRIFWRRQVHLAAGIRPNTITSSFPQLMSNVDAAYEVAERGIAFFFKGPHYWITRGFQMQGPPRIIYDFGFPRHVQRIDAAVYLRKPQKTLFFVGDEYYS?????
???

>Microtus
MKVLPASGLAVLVTALKFATAVPTLFAATPRTFRSSYQLAQDYLDKYYTPKGGPQAGEMVVRQGNPMVRKIKELQSFFGLQVTGKLDPTTMHVIKRPRCGVPDVA
NYRLFPGEPKWKKNILTYRVSKYTPSMSPAEVDKAVEMALRAWSTAVPLNFVRINSGEADIMISFETGDHGDSYPFDGPRGTLAHAFAPGEGLGGDTHFDNAEKW
TMGMNGFNLFTVAAHEFGHALGLGHSTDPSALMYPTYKYQNPYRFHLPKDDVKGIQALYGPRKTFPGKPTMPHIPPHKPSLPDLCDSSSSFDAVTMLGKELLLFK
DRIFWRRQVHLAAGIRPNTITSSFPQLMSNVDAAYEVPERGLAFFFKGPHYWITRGFQMQGPPRTIYDFGLPRRVQRIDAAVYLRKPQKTLFFVGEEYYSYDERK
KKMEKDYPKNTEEEFSGVSGHVDAAVELNGYIYFFSGPKTFKYDTEKEDVVSVVKSSSWIGC*
>Jaculus
MKALPASGLAVLLVTALKFSAAAPTLFAATPRTWRNNYHLAQAYLDKFYTKKGGHQVGELAVRGGNSMVKKIKELQTFFGLQVTGKLDQNTMDVIKRPRCGVPDV
ANYRLFPGEPKWKKNMLTYRISKYTPSMRPAEVDKAVEMALRAWSSAVPLNFVRTHAGEADIMISFESGDHGDSYPFDGPRGTLAHAFAPGEGLGGDTHFDNAEK
WTMGTNGFNLFTVAAHEFGHALGLAHSTDPSALMYPTYKYQHPFGFRLPKDDVKGIQALYGPRKTFPGKPTMPHVPSHKPSIPDLCDSSSSFDAVTMLGKELLFF
KDRIFWRRQVHLPSGIRPSTITSSFPQLMSNVDAAYDVAERGIAFFFKGPHYWITRGFQMQGPPRTIYDFGFPRHVQRIDAAVYLKEPQKTLFFVGDEYYSFDER
KRKMEKDYPKTTEEEFSGVSGQIDAAVELNGYIYFFSGPKTFKYDTEKEDVVSVVKSSSWVGC*
>Dipodomys
MKVLPASGLAVLLVTALKLSTAFPSLFVATPRTWRNNYHLAQAYLQKYYTSKGNLQMGEMVARGGSPLVKKVKELQAFLGLQVTGKLDRNTMDMIKKPRCGVPDV
ANYRLFPGEPKWKKNTLTYRISKYTPSMRPAEVDKAVEMALRAWSSAVPLNFVRINSGEADIMISFETGDHGDSYPFDGPRGTLAHAFAPGEGLGGDTHFDNAEK
WTMGTNGFNLFTVAAHEFGHALGLAHSTDPSALMYPTYKYQQPYGFRLPKDDVKAIQPLYGLRKPFLGKPTAPHIPPHKPSIPDLCDSSSSFDAVTMLGKELLFF
KDRIFWRRQVHLPSGIRPSTITSSFPQLMSNVDAAYEVAERGLAFFFK???YDER
KRKMEKDYPKSTEEEFSGVNGQIDAAVELNGYIYFFSGPKTFKYDTEKEDVVSVLKSSSWVGC*
>Cavia
MKVLPASGLAVLLLTALKFSSAAPTLFAATPRTWRNNFHLAQAYLDKYYTKKGGHQVGEMVSRGGNSMVKKIKELQTFFGLPLTGKLDQSTMDVIKKPRCGVPDV
ANYRLFPGEPKWEKNTLTYRISKYAASMSPAEVERAIEMALRAWSSAVPLNFVRINSGEADIMVSFETGDHGDSYPFDGPRGTLAHAFAPGEGLGGDTHFDNAEK
WTLGMNGFNLFTVAAHEFGHALGLAHSTDPSALMYPTYKYKNPYGFRLPKDDVKGIQALYGPRKPYLGKPTVPHLPPRKPSIPDVCDSSSSFDAVTMLGKELLFF
KDRIFWRRQVHLLSEIRPRTITSSFPQLMSNVDAAYEVAERGTAYFFKGPHYWVTRGFQIQGPPRTIYDFGFPRHVQRIDAAVYLKEPRKTLFFVGDEYYSYDER
KRKMEKDYPKSTEEEFSGVNGQIDAAVELNGYIYFFSGPKTYRYDIEKEDVVSVVKSSSWIGC*
>Octodon
MKVLPASGLAVLLVTALEFSSATPSLFAATPRTWRNNFHLAQAYLEKYYMKRGGHQAGEMVSRSSMARRIKDLQAFLGLRVTGKLDQRTMDTIKQPRCGVPDVAN
YRLFPGEPKWEKNTLTYRVAKYAASMRPAEVDRAIEMALRAWSSAVPLNFVRIHSGEADIMVSFETGDHGDSYPFDGPRGTLAHAFAPGEGLGGDTHFDNAEKWT
LGMNGFNLFTVAAHEFGHALGLAHSTDPSALMYPTYKYKNPYGFRLPKDDVKGIQALYGPRKPFLGKPMGPHLPPHKPSIPDVCDSSSSFDAVTMLGKELLLFKD
RIFWRRQVHLLAEIRASTITSSFPQLMSNVDAAYEVAERGTAYFFKGPHYWITRGFHIQGPPRTIYDFGFPRDVQRIDAAVYLKEPQKTLFFVGDEYYSFDERKR
KMEKDYPKSTEEEFSGVHGQVDAAVELNGYIYFFSGPKTYKYDIEKEDVVSVVKSSSWIGC*
>Chinchilla
MKALPASGLAVLLVTALKFSSAAPLLAATPRTWRNNFHLAQAYLDKYYTKRGGHQVGEMVSRGGNSMVKKIKELQAFLGLQVTGKLDQNTMDVIKMPRCGVPDMA
NYRLFPGEPKWEKNTLTYRISKYAASMRPAEVDRAIEMALRAWSSAVPLNFVRVNSGEADIMVSFETGDHGDSYPFDGPRGTLAHAFAPGEGLGGDTHFDNAEKW
TLGMNGFNLFTVAAHEFGHALGLAHSTDPSALMYPTYKYKNPYGFRLPKDDVKGIQALYGPRKPFLGKPTIPHIPPHKPSFPDVCDASSSFDAVTMLGKELLLFK
DRIFWRRQVHLLAEIRPSTITSSFPQLMSNVDAAYEVAERGTAYFFKGPHYWITRGFQIQGPPRTIYDFGFPRRVQRIDAAVYLQEPQKTLFFVGDEYYSYDERK
RKMEKDHPKSTEEEFSGVHGQIDAAVELNGYIYFFSGPKTYKYDVEKEDVVSVVKSSSWIGC*
>Fukomys
MKMLPASGLAVLLVTALKFSSTAPTLFAATPRTWRNNFHLAQAYIDKYYTKKDGHQVGEMVSRGGNPMAKKIKELQAFFGLQVTGKLDQNTMDVIKKPRCGVPDV
ANYRLFPGEPKWEKNILTYRISKYASSMSPAEVDRAIEMALQAWSSAVPLNFVKISSGEADIMVSFETGDHGDSYPFDGPRGTLAHAFAPGEGLGGDTHFDNAEK
WTLGMNGFNLFTVAAHEFGHALGLAHSTDPSALMYPTYKYKNPYGFRLPKDDVKGIQALYGPRKPFLGNPTLPHGPPHKPSIPDICDSSSSFDAVTMLGKELLFF
KDRIFWRRQVHLLAEIRPSTITSSFPQLMSNVDAAYEVAERGVAYFFKGPHYWITRGFQIQGPPRTIYDFGFPRHVQRIDAAVYLKEPQKTLFFVEDEYYSYDER
KRKMEKDYPKSTEEEFSGVHGQIDAAVELNGYIYFFSGPKTYKYDIEKEDVVGVVKSSSWIGC*
>Heterocephalus
MKVLPASGLAVLLVTALKFSSADPTVFAATPRTWRNNFHLAQAYLDKYYTKKGSLQVGEMVSRGGNPMVKKIKELQAFFGLQVTGKLDQNTMDVIKKPRCGVPDV
ANYRLFPGEPKWEKNTLTYRISKYASSMSPAEVDRAIQMALQAWSSAVPLNFVRINSGEADIMVSFETGDHGDSYPFDGPRGTLAHAFAPGEGLGGDTHFDNAEK
WTLGMNGFNLFTVAAHEFGHALGLAHSTDPSALMYPTYKYKNPYGFRLPKDDVKGIQALYGPRKPFLGKPTIPHVPPHKPSIPDVCDSLSSFDAVTMLGKELLFF
KDRIFWRRQVHLLAEIRPSTITSSFPQLMSNVDAAYEVAERGVAYFFKGPHYWITRGFQIQGPPRTIYDFGFPRRVQRIDAAVYLKKPQKTLFFVGDEYYSYDER
KRKMEKDYPKSTEEEFSGVHGQIDAAVELNGYIYFFSGPKTYKYDIEKEDVVSVVKSSSWIGC*
>Nannospalax
MKVLPASGLAVLLVTALKFATAAPTLFAATLRSSRNNHHLAQAYLDKYYTKNGVHQAGEMVARGGNSMVKKIKELQTFFGLQVTGKLDQNTMDVIKRPRCGVPDV
ANYRLFPGEPKWKKNILTYRVSKYTSSMSPPEVDKAIEMALRAWSTAVPLSFIRVNSGEADIMISFETGDHGDSYPFDGPRGTLAHAFAPGEGLGGDTHFDNAEK
WTMGMNGFNLFTVAAHEFGHALGLAHSTDPSALMYPTYKYQNPFRFHLPKDDVKGIQALYGPRKTFPGKPTVPHVPPHTPSIPDLCDSSSSFDAVTMLGNELLFF
KDRIFWRRQVHVPAGIRPSTITSSFPQLMSNVDAAYEMAERGIAFFFKGPHYWITRGFQMQGPPRTIYDFGFPRHVQRIDAAVYLKEPQKTLFFVGDEYYSYDER
KRKMEKDYPKNTEEEFSGVSGQIDAAVELNGYIYFFSGPKTFKYDMEKEDVVSVVKSSSWVGC*
>Ictidomys
MKVLPASGLAVLLITALKFSTAAPSLFAATPRTWRNNYHLAQEYLDKYYTKKGGHPVGEMAARGGNAMVKKIKELQAFFGLQVNGKLDQNTMDVIKRPRCGVPDV
ANYRLFPGEPKWKKNTLTYRIAKYTSSMRPIEVEKAVEMALQAWSSAVPLSFVRINSGEADIMISFETGDHGDSYPFDGPRGTLAHAFAPGEGLGGDTHFDNAEK
WTMGMNGFNLFTVAAHEFGHALGLAHSSDPTALMYPTYKYQNPYGFRLPKDDVKGIQALYGPRKPFLGKPTIPHVPPHKPSNPDPCDSRASFDAVTMLGKELLFF
RDRIFWRRQVHVPAGIRPSTITSSFPQLMSNVDAAYEVAERGTAYFFKGPHYWITRGFQMQGPPRTIYDFGFPRHVQRIDAAVYLKKPQKTLFFVGDEYYSYDER
KRKMDKDYPKNTEEEFSGVSGQIDAAVELNGHIYFFSGPKTYKYDTEKEDVVSVVKSSSWIGC*
>Oryctolagus
MKVLPASGLAVLLVTALKFSTATPSLFAATPRTWRNNFHLAQAYLDKYYTRKGGSQIGEMVARGGNSMVKKIKELQAFFGLRVTGKLDQNTMDVIKKPRCGVPDV
ANYRLFPGEPKWKKNTLTYRVAKYTSSMTPFEVDKAIEMALRAWSSAVPLSFVRVNAGEADIMISFETGDHGDSYPFDGPRGTLAHAFAPGEGLGGDTHFDNAEK
WTMGTNGFNLFTVAAHEFGHALGLAHSTDPSALMYPTYKYQNPFGFRLPKDDVKGIQALYGPRKPFLGKPHMPHIPPHKPSIPDLCDSSSSFDAVTMLGKELLFF
RDRIVWRRQAHLPTGVQPSTITSSFPQLMSNVDAAYEVADRGTAYFFKGPHYWITRGFQMLGSPRTIYDFGFPRYVQQIDAAVYLKEPQKTLFFVGDEYYSYDER
KRKMEKDYPKSIEEEFSGVNGQIDAAMELNGYIYFFSGPKAYKYDTEKEDVVSVMKSNSWIGC*
>Ochotona
MKVLPASGLAVFLIMALKFSAAAPSLFTATPRTWRNNFHLAQAYLDKYYTKKGGTQVGEMVARGGNAMVKKIKELQAFFGLQVTGKLDQNTMDVIKKPRCGVPDV
ANYRLFPGEPKWKKNTLTYRVSKYTSSMAPVEVDKAVEMALRAWSSAVPLNFVRVNTGEADIMISFETGDHGDSYPFDGPRGTLAHAFAPGEGLGGDTHFDNAEK
WTMGTNGFNLFTVAAHEFGHALGLAHSTDPTALMYPTYKYQNPYGFHLPKDDVRGIQALYGPRKPFLGKPTLPHIPPHKPILPDLCDSSSSFDAVTMLGKELLFF
RDRIFWRRQLHLPTGVQPSTITSSFPQLMSNVDAAYEVAERGTAYFFKGPHYWVTRGFQMQGSPRTIYDFGFPRYVQRIDAAVFLKEPQKTLFFVGDEYYSFDEQ
KRKMEKDYPKSIEEEFSGVNGQIDAAVELNGYIYFFSGPKTYKYDTEKEDVVSVMKSNSWIGC*

Cetartiodactyla
>Bos
MKMLPASGLAVLLVTALKFSTAAPSLPAASPRTSRNNYRLAQAYLDKYYTKKGGPQIGEMVARGGNSTVKKIKELQEFFGLRVTGKLDRATMDVIKRPRCGVPDV
ANYRLFPGEPKWKKNTLTYRISKYTPSMTPAEVDRAMEMALRAWSSAVPLNFVRINAGEADIMISFETGDHGDSYPFDGPRGTLAHAFAPGEGLGGDTHFDNAEK
WTMGTNGFNLFTVAAHEFGHALGLAHSTDPSALMYPTYKYQNPYGFRLPKDDVKGIQALYGPRRAFSGKPTAPHGPPHNPSIPDLCDSNLSFDAVTMLGKELLLF
RDRIFWRRQVHLMSGIRPSTITSSFPQLMSNVDAAYEVAERGTAYFFKGPHYWITRGFQMQGPPRTIYDFGFPRYVQRIDAAVYLKDAQKTLFFVGDEYYSYDER
KRKMEKDYPKSTEEEFSGVNGQIDAAVELNGYIYFFSGPKAYKYDTEKEDVVSVLKSSSWIGC*

>Capra
MKVLPASGLAVLLITALKFSTAAPSLLAASPRTSRNNYRLAQAYLDKYYTRKGGPQIGEMVARGGNSTVKKIKELQEFFGLRVTGKLDQATMDVIKRPRCGVPDV
ADYRLFPGEPKWKKNTLTYRISKYTPSMTPAEVDRAMEMALRAWSSAVPLNFVRVNAGEADIMISFETGDHGDSYPFDGPRGTLAHAFAPGEGLGGDTHFDNAEK
WTMGTNGFNLFTVAAHEFGHALGLAHSTDPSALMYPTYKYQNPYGFHLPKDDVKGIQALYGPRRAFPGKPTAPHGPPHNPSIPDLCDSNLSFDAVTMLGKELLLF
RDRIFWRRQVHLMSGIRPSTITSSFPQLMSNVDAAYEVADRGTAYFFKGPHYWITRGFQMQGPPRTIYDFGFPRYVQQIDAAVYLKDARKTLFFVGDEYYSYDER
KRKMEKDYPKSTEEEFSGVNGQIDAAVELNGYIYFFSGPKAYKYDTEKEDVVSVLKSSSWIGC*
>Ovis
MKVLPASGLAVLLITALKFSTAAPSLLAASPRTSRNNYRLAQAYLDKYYTRKGGPQIGEMVARGGNSTVKKIKELQEFFGLRVTGKLDRATMDVIKRPRCGVPDV
ADYRLFPGEPKWKKNTLTYRISKYTPSMTPAEVDRAMEMALRAWSSAVPLNFVRVNAGEADIMISFETGDHGDSYPFDGPRGTLAHAFAPGEGLGGDTHFDNAEK
WTMGTNGFNLFTVAAHEFGHALGLAHSTDPSALMYPTYKYQNPYGFHLPKDDVKGIQALYGPRRAFPGKPTAPHGPPHNPSIPDLCDSNLSFDAVTMLGKELLLF
RDRIFWRRQVHLMSGIRPSTITSSFPQLMSNVDAAYEVADRGTAYFFKGPHYWITRGFQMQGPPRTIYDFGFPRYVQQIDAAVYLKDAQKTLFFVGDEYYSYDER
KRKMEKDYPKSTEEEFSGVNGQIDAAVELNGYIYFFSGPKAYKYDTEKEDVVSVLKSSSWIGC*
>Pantholops
MKVLPASGLAVLLITALKFSTAAPSLLAASPRTSRNNYRLAQEYLDKYYTKKGGPQIGEMVARGGNSTVKKIKELQEFFGLRVTGKLDRATLDVIKRPRCGVPDV
ADYRLFPGEPKWKKNTLTYRISKYTPSMTPAEVDRAMEMALRAWSSAVPLNFVRVNAGEADIMISFETGDHGDSYPFDGPRGTLAHAFAPGEGLGGDTHFDNAEK
WTMGTNGFNLFTVAAHEFGHALGLAHSTDPSALMYPTYKYQNPYGFHLPKDDVKGIQALYGPRRAFPGKPTAPHGPPHNPSIPDLCDSNLSFDAVTMLGKELLLF
RDRIFWRRQVHLMSGIRPSTITSSFPQLMSNVDAAYEVADRGTAYFFKGPHYWITRGFQMQGPPRTIYDFGFPRYVQQIDAAVYLKDARKTLFFVGDEYYSYDER
KRKMEKDYPKSTEEEFSGVNGQIDAAVELNGYIYFFSGPKAYKYDTEKEDVVSVLKSSSWIGC*
>Tursiops
MKVFPASGLAVLLVTALKFSTAAPSLFAATPRTSRNSYHLAQAYLDKYYTKKGGHQIGEMIARGGNSMVKNIKELQAFFGLRVTGKLDRATMDVIKRPRCGVPDV
ANYRLFPGEPKWKKNTLTYRISKYTPSMTSAEVDKAVEMALQAWSSAVPLSFVRINSGEADIMISFETGDHGDSYPFDGPRGTLAHAFAPGGGLGGDTHFDNAEK
WTMGMNGFNLFTVAAHEFGHALGLAHSTDPSALMYPTYKYQHPYGFHLPKDDVKGIQALYGPRKTFPGKPAVPHSPPHNPSIPDLCDSSSAFDAVTMLGKELLFF
RDRIFWRRQVHLMSGIRPNTITSTFPQLISNVDAAYEVAERGTAYFFKGPHYWITRGFQMQGTPRTIYDFGFPRYVQRIDAAVYLKDAQKTLFFVGDEYYSYDER
KRKMEKDYPKNTEEEFSGVNGQIDAAMELNGYIYFFSGPKAYKYDTEKEDVVSVLKSSSWIGC*
>Orcinus
MKVLPASGLAVLLVTALKFSTAAPSLFAATPRTSRNSYHLAQAYLDKYYTKKGGHQIGEMIARGGNSMVKNIKELQAFFGLRVTGKLDRATMDVIKRPRCGVPDV
ANYRLFPGEPKWKKNTLTYRISKYTPSMTSAEVDKAVEMALQAWSSAVPLSFVRINSGEADIMISFETGDHGDSYPFDGPRGTLAHAFAPGEGLGGDTHFDNAEK
WTMGMNGFNLFTVAAHEFGHALGLAHSTDPSALMYPTYKYQHPYGFHLPKDDVKGIQALYGPRKTFPGKPAVPHSPPHNPSIPDLCDSSSAFDAVTMLGKELLFF
RDRIFWRRQVHLMSGIRPNTITSSFPQLMSNVDAAYEVAERGTAYFFKGPHYWITRGFQMQGTPRTIYDFGFPRYVQRIDAAVYLKDAQKTLFFVGDEYYSYDER
KRKMEKDYPKNTEEEFSGVNGQIDAAMELNGYIYFFSGPKAYKYDTEKEDVVSVLKSSSWIGC*
>Lipotes
MKVLPASGLAVLLVTALKFSTAAPSLFAATPRTSRNSYHLAQAYLDKYYTKKGGHRIGEMIARGGNSMVKNIKELQAFFGLRVTGKLDRATMDVIKRPRCGVPDV
ANYRLFPGEPKWKKNILTYRISKYTPSMTSAEVDKAVEMALQAWSSAIPLSFVRINSGEADIMISFETGDHGDSYPFDGPRGTLAHAFAPGEGLGGDTHFDNAEK
WTMGMNGFNLFTVAAHEFGHALGLAHSTDPSALMYPAYKYQHPYGFHLPKDDVKGIQALYGPRKTFPGKPAVPHSPPHNPSIPDLCDSSSAFDAVTMLGKELLFF
RDRIFWRRHVHLMSGIQPSTITSSFPQLMSNVDAAYEVAERGTAYFFKGPHYWITRGFQMQGTPRTIYDFGFPRYVQRIDAAVYLKDAQKTLFFVGDEYYSYDER
KRKMEKDYPKNTEEEFSGVNGQIDAAMELNGYIYFFSGPKAYKYDTEKEDVVSVLKSSSWIGC*
>Physeter
MKVIPASGLAVLLITALKFSTAAPSLFAATPRTSRNSYHLAQAYLDKYYTKKGGHQIGEMIARGGNSMVKNIKELQAFFGLRVTGKLDRATMDVIKRPRCGVPDV
ANYRLFPGEPKWKKNPLTYRISKYTPSMTSAEVDKAVEMALQAWSSAVPLNFVRINSGEADIMISFETGDHGDSYPFDGPRGTLAHAFAPGEGLGGDTHFDNAEK
WTMGMNGFNLFTVAAHEFGHALGLAHSTDPSALMYPTYKYQHPYGFHLPKDDVKGIQALYGPQKTFPGKPAVPHSPPHNPSIPDLCDSSSAFDAVTMLGKELLFF
RDRIFWRWQVHLMSGIRPSTITSSFPQLMSNVDAAYEVAERGTAYFFKGPHYWITRGFQMQGSPRTIYDFGFPRYVQRIDAAVYLKDAQKTLFFVGDEYYSYDER
KRKMEKDYPKNTEDEFSGVNGQIDAAVELNGYIYFFSGPKAYKYDREKEDVVSVLKSSSWIGC*
>Vicugna
MKVLPASGLAVLLVTALKFSSAAPSLFAATPRTSRNNYHLAQAYLDKYYTKKGGHPLGEMAARGSNSMVKKIKELQQFFGLRVTGKLDRATMDVIKRPRCGVPDV
ANYRLFPGEPKWKKNTLTYRISKYTPSMTPAEVDRAVQMALQAWSSAVPLSFVRVNAGEADIMISFETGDHGDSYPFDGPRGTLAHAFAPGEGLGGDTHFDNAEK
WTMGTNGFNLFTVAAHEFGHALGLAHSTDPSALMYPTYKYQRPYGFHLPKDDVKGIQALYGPRKTFPGKPTVPHASPHNPSIPDLCDSGSSFDAVTMLGKELLFF
RDRIFWRRQVHLMSGIRPSTITSSFPQLMSNVDAAYEVADRGTAYFFKGPHYWVTRGFQMQGPPRTIYDYGFPRNVQRIDAAVYLKDAQKTLFFVGDEYYSYDER
KRKMEKDYPKITEEEFSGVNGQIDAAVELNGNIYFFSGPKAYKYDTEKEDVVSVLRAGSWVGC*
>Camelus
ferusMKVLPASGLAVLLVTALKFSSAAPSLFAATPRTSRNNYHLAQAYLDKYYTKKGGHPLGEMAARGGNSMVKKIKELQQFFGLRVTGKLDRATMDVIKRPRC
GVPDVANYRLFPGEPKWKKNTLTYRISKYTPSMTPAEVDRAVQMALQAWSSAVPLSFVRVNAGEADIMISFETGDHGDSYPFDGPRGTLAHAFAPGEGLGGDTHF
DNAEKWTMGTNGFNLFTVAAHEFGHALGLAHSTDPSALMYPTYKYKHPYGFHLPKDDVKGIQALYGPRKTFPGKPTVPHASPHNPSIPDLCDSGSSFDAVTMLGK
ELLFFRDRIFWRRQVNLMSGIRPSTITSSFPQLMSNVDAAYEVADRGTAYFFKGPHYWVTRGFQMQGPPRTIYDYGFPRNVQRIDAAVYLKDAQKTLFFVGDEYY
SYDERKRKMEKDYPKNTEEEFSGVNGQIDAAVELNGNIYFFSGPKAYKYDTEKEDVVSVLRAGSWVGC*
>Sus
MKVLPASGLAVLLVTALKFSAAAPSLFAATPRTSRNNYHLAQAYLDKYYTKKGGHQVGEMVAKGGNSMVKKIKELQAFFGLRVTGKLDRTTMDVIKRPRCGVPDV
ANYRLFPGEPKWKKNTLTYRISKYTPSMTPAEVDKAMEMALQAWSSAVPLSFVRVNAGEADIMISFETGDHGDSYPFDGPRGTLAHAFAPGEGLGGDTHFDNAEK
WTMGMNGFNLFTVAAHEFGHALGLAHSTDPSALMYPTYKYQNPYGFHLPKDDVKGIQALYGPRKTFTGKPTVPHGPPHNPSLPDICDSSSSFDAVTMLGKELLFF
RDRIFWRRQVHLMSGIRPSTITSSFPQLMSNVDAAYEVADRGMAYFFKGPHYWITRGFQMQGPPRTIYDFGFPRYVQRIDAAVHLKDTQKTLFFVGDEYYSYDER
KRKMDKDYPKNTEEEFSGVNGQIDAAVELNGYIYFFSGPKAYKYDTEKEDVVSVLKSNSWIGC*

Perissodactyla
>Equus
MKVLLASGLAVLLITALKFSTATPSLFAATPRTSRNNYHLAQAYLDKYYTVKGGHQIGEMVARGSNSMVKKIKELQVFFGLPVTGKLDKSTMDVIKRPRCGVPDV
ANYRLFPGEPKWKKNTLTYRISKYTTSMTPADVNKAVEMALQAWSSAVPLNFVRVDSGEADIMISFETGDHGDSYPFDGPRGTLAHAFAPGEGLGGDTHFDNAEK
WTMGTNGFNLFTVAAHEFGHALGLAHSTDPSALMYPTYKYQNPYGFHLPKDDVKGIQALYGPRRAFLGKPTMPHAPPRNPSIPDLCDSGSSFDAVTMLGKELLFF
RDRIFWRRQIHLAAGIRPSMITSSFPQLMSNVDAAYEVADRGTAYFFKGPHYWITRGFQIQGPPRTIYDFGFPRYVQRIDAAVHLKDAQKTLFFVGDEYYSYDER
KRKMEKDYPKSTEEEFSGVNGQIDAAVELNGYIYFFSGPKAYKYDTEKEDVVSVLKSSSWIGC*
>Ceratotherium
MKVFLASGLAVLLITALKFSTATPSLFAATPRTSRNNYHLAQAYLDKYYTVKGGHQVGEMVARGSKSMVKKIKELQVFFGLPVTGKLDKPTMDVIKRPRCGVPDV
ANYRLFPGEPKWKKNTLTYRISKYTTSMTSADVDKAVQMALQAWSSAVPLSFVRVDSGEADIMISFETGDHGDSYPFDGPRGTLAHAFAPGEGLGGDTHFDNAEK
WTMGMNGFNLFTVAAHEFGHALGLAHSTDPSALMYPTYKYQNPYRFHLPKDDVKGIQALYGPRKIFPGKPTMPHAPHHNPSIPDLCDSSSSFDAVTMLGKELLFF
RDRIFWRRQIHLAAGIRPSMITSSFPQLMSNVDAAYEVADRGTAYFFKGPHYWITRGFQMQGPPRTIYDFGFPRYVQRIDAAVYLKDAQKTLFFVGDEYYSYDER
KRKMEKDYPKYTEEEFSGVNGQIDAAVELNGYIYFFSGPKAYKYDTEKEDVVSVLKSSSWIGC*

Carnivora
>Canis
MTVLPMCGLALLLGAALEFCTAAPSVSAAAPRTTQNKYHLAQAYLDKYYTSKAGPQVGEMGAPGGRALIKKIKELQAFFGLRITGKLDRPTMDMIKRPRCGVPDV
ANYRLFPGEPKWKKNTLTYRISKYTSSMSPAEVDKAVEMALQAWGSAVPLSFIRVNSGEADIMISFETGDHGDSYPFDGPRGTLAHAFAPGEGLGGDTHFDNAEK
WTMGMNGFNLFTVAAHEFGHALGLAHSTDPSALMYPTYKYQHPYGFHLPKDDVKGIQALYGPRKTLLGKPTVPHAPPQSPSIPDLCDSSSSFDAVTMLGKELLLF
RDRIFWRRQVHLMAGIRPSTITSSFPQLMSNVDAAYEVAERGTAYFFKGPHYWITRGFQMQGPPRTIYDFGFPRYVQRIDAAVYLKDVQKTLFFVGDEYYSYDER
KRKMEKDYPKNTEEEFSGVNGQIDAAVELNGYIYFFSGPKAYKYDTEKEDVVSVLKSSSWIGC*

>Ailuropoda
MKVLPASGLAVLLIAALKFSTAAPSLLADTPGTARNNFHLAQAYLDKYYTRKAGPQVGELGAPGGRALVKKIKELQAFFGLRVTGKLDRRTMDVIKRPRCGVPDV
ANYRLFPGEPKWKKNTLTYRISKYTPSMTPAEVDKAVEMALQAWSSAVPLGFVRVNAGEADIMISFETGDHGDSYPFDGPRGTLAHAFAPGEGLGGDTHFDNAEK
WTMGMNGFNLFTVAAHEFGHALGLAHSTDPSALMYPTYKYQNPYGFHLPKDDVKGIQALYGPRKPLLGKPTVPHAPPQNPSNPDLCDSSFSFDAVTMLGKELLLF
RDRIFWRRQVHVMAGIRPSTITSSFPQLMSNVDAAYEVADRGTAYFFKGPHYWITRGFQMQGPPRAIYDFGFPRDVQRIDAAVYLKDVQKTLFFVGDDYYSYDER
KRKMEKDYPKNTEEEFSGVNGQIDAAVELNGYIYFFSGPKAYKYDTEKEDVVSVLKSSSWIGC*
>Ursus
MKVLPASGLAVLLIAALKFSTAAPSLFAATPGTARNNFHLAQAYLDKYYTRKAGPQVGEMGAPGGRALVKKIKELQAFFGLRVTGKLDRRTMDVIKRPRCGVPDV
ANYRLFPGEPKWKKNTLTYRISKYTPSMAPAEVDKAVEMALQAWSSAVPLGFVRVNAGEADIMISFETGDHGDSYPFDGPRGTLAHAFAPGEGLGGDTHFDNAEK
WTMGMNGFNLFTVAAHEFGHALGLAHSTDPSALMYPTYKYQNPYGFHLPKDDVKGIQALYGPRKPFLGKPTVPHAPPQNPSNPDLCDSSFSFDAVTMLGKELLLF
RDRIFWRRQVHVMAGIRPSTITSSFPQLMSNVDAAYEVADRGTAYFFKGPHYWITRGFQMQGPPRTIYDFGFPRYVQRIDAAVYLKDVQKTLFFVGDEYYSYDER
KRKMEKDYPKNTEEEFSGVSGQIDAAVELNGYIYFFSGPKAYKYDTEKEDVVSVLKSSSWIGC*
>Leptonychotes
MKVLPASGLALLLTTALKFSTAAPSLFAATPRTSRNNYRLAQAYLDKYYTRKAGPQVGEMGAPGGRALVKKIKELQAFFGLRVTGRLDRPTMDVIKRPRCGVPDV
ANYRLFPGEPKWKKNTLTYRISKYTPSMTSAEVDRAVEMALQTWSSAVPLSFARVNAGEADIMISFETGDHGDSYPFDGPRGTLAHAFAPGEGLGGDTHFDNAEK
WTMGMNGFNLFTVAAHEFGHALGLAHSTDPSALMYPTYKYQHPYGFHLPKDDVKGIQALYGPRKTFLGKPTVPHAPPQNPSKPDLCDSSSPFDAVTMLGKELLLF
RDRIFWRRQVHMMAGIRPSTITSSFPQLMSNVDAAYEVSDRGTAYFFKGPHYWITRGFQMQGPPRTIYDFGFPRYVQRIDAAVYLKDVQKTLFFVGDEYYSYDER
KRKMEKDYPKNTEEEFSGVNGQIDAAVELNGYIYFFSGPKAYKYDTEKEDVVSVLKSSSWIGC*
>Odobenus
MKVLPTSGLALLLITALKFSTAAPSLFAATPRTSRNNYHLAQAYLDKYYTRKAGPQVGEMGAPGGRALVKKIKELQAFFGLRVTGKLDRPTMDVIKRPRCGVPDV
ANYRLFPGEPKWKKNTLTYRISKYTPSMTSAEVDKAVEMALQAWSSAVPLSFVRVNTGEADIMISFETGDHGDSYPFDGPRGTLAHAFAPGEGLGGDTHFDNAEK
WTMGTNGFNLFTVAAHEFGHALGLAHSTDPSALMYPTYKYQHPYGFHLPKDDVKGIQALYGPRKTFLGKPTVPHARPQNPSKPDLCDSSSPFDAVTMLGKELLLF
RDRIFWRRQVHMMAGIRPSTITSSFPQLMSNVDAAYEVADRGTAYFFKGPHYWITRGFQMQGPPRTIYDFGFPRYVQRIDAAVYLKDVQKTLFFVGDGYYSYDER
KRKMEKDYPKNTEEEFSGVNGQIDAAVELNGYIYFFSGPKAYKYDTEKEDVVSVLKSSSWIGC*
>Mustela
MKVLPAPGLALLLLTALELSATAPSLSAATPGTSRNNYHLAQAYLDKYYTREAGPQVGEMGAAGGRALVKKIKELQAFFGLRVTGKLDRPTMDVIKRPRCGVPDV
ANYRLFPGEPKWKKNSLTYRISKYTPSMPSAEVDKAVEMALQAWSSAVPLSFVRVNAGEADIMISFETGDHGDSYPFDGPRGTLAHAFAPGEGLGGDTHFDNAEK
WTMGMNGFNLFTVAAHEFGHALGLAHSTDPSALMYPTYKYQHPYGFHLPKDDVKGIQALYGPRKTFLGKPTMPHAPPQSPSNPDLCDSSSSFDAVTMLGKELLLF
RDRIFWRRQVHVMAGIRPSTITSSFPQLMSNVDAAYEVADRGTAYFFKGPHYWITRGFQMQGPPRTIYDFGFPRYVQRIDAAVYLKDVQKTLFFVGDEYYSYDER
KRKMEKDYPKNTEEEFSGVNGQIDAAVELNGYIYFFSGPKAYKYDTEKEDVVSVLKSSSWIGC*
>Felis
MKVLPASGLAVLLVTALKFSTAAPSPPAASPRTSRNNYHLAQAYLDKYYTRKGGHQLGEMAARGASSLVKKIKELQAFFGLRVTGKLDRSTMDVIKRPRCGVPDV
ANYRLFPGEPKWKKNTLTYRISKYTPSMAAADVDKAVEMALQAWGSAVPLTFVRVTSGEADIMISFESGDHGDSYPFDGPRGTLAHAFAPGEGLGGDTHFDNAEK
WTMGMNGFNLFTVAAHEFGHALGLAHSTDPSALMYPTYKYQHPYGFHLPKDDVKGIQALYGPRKTFPGKPTVPHAPPQSPSTPDLCDSSSSFDAVTMLGKELLFF
RDRIFWRRQVHMMAGIRPSTITSSFPQLMSNVDAAYEVAERGTAYFFKGPHYWVTRGFQMQGPPRTIYDFGFPRYVQRIDAAVYLKDVQKTLFFVGDEYYSYDER
KGKMEKDYPKNTEEEFSGVNGQIDAAVELNGYIYFFSGPKAYKYDTEKEDVVSVLKSSSWIGC*

Eulipotyphla
>Erinaceus
MKVLPTSGFAVLLIMALKLSTAAPSLFAATPRTSRNNYHLAQAYLDRYYTKKEEYQIGEMVARGHNSMIKKIKELQAFFGLQITGKLDRTTMDVIKKPRCGVPDV
ANYRLFPGEPKWKKNTLTYRISKYTSSMTSAEVDKAVEMALQAWSSAVPLNFVKINSGEADIMISFETGDHGDSYPFDGPRGTLAHAFAPGEGLGGDTHFDNAEK
WTMGMNGFNLFTVAAHEFGHALGLAHSTDPSALMYPTYKYQHPYGFHLPKDDVKGIQALYGPRKTFPGKPTIPFSPPHNPSIPDLCDTSSSFDAVTMLGKELLFF
RDRIFWRRQVHLPGGIRPSTITSSFPQLMSNVDAAGEVAERGTAYFFKGPHYWITRGFRMQGPPRTIYDFGFPRYVQRIDAAVYLKDAQKTLFFVGDEYYSYDER
KGKMEKDYPKNTEEEFSGVNGQIDAAVELNGYIYFFSGPKAYKYDTEKEDVVSVVKSSSWIGC*
>Sorex
MKVLPASGFAVLLFATLKFSTAASSLLAAAPRTSWNNYHLAQAYLNKYYTKEGGHQIGELVARRGNSMVKKIKELQTFFNLQVTGKLDKSTMDVIKRPRCGVPDV
ANYRLFPGEPKWKKNTLTYRISKYTSSMTPAEVDKAVEMALQAWSSAVPLSFVRVNSGEADIMISFETGDHGDSYPFDGPRGTLAHAFAPGEGLGGDTHFDNAEK
WTMGTNGFNLFTVAAHEFGHALGLAHSSDPSALMYPTYKYQRPYGFHLPKDDVKGIQALYGPRKPFLGKPTSPHTPPHHPDLCDSTSSFDAVTMLGKELLFFRDR
IFWRRQVHLPSGIRPSTITSSFPQLMSNVDAAYEVAERGTAYFFKGPHYWITRGFQMQGPPRTIYDFGFPRYVQRIDAAVYVPDTQKTLFFVGDEYYSYDERKGK
MEKDYPKNTEEEFPGVNGQIDAAVELNGYIYFFSGPKAYKYDVEKEDVVSVVKSSSWIGC*
>Condylura
MKVLPASGLAVLLITALKLSTAAPSLFAATHRTSRNNYHLAQAYLDKYYTKKGGQQIGEMVARGSNSMVKKIKELQAFFGLQITGKLDRSTMDMIKKPRCGVPDV
ANYRLFPGEPKWKKNTLTYRISKYTSSMTSAEVDKAVEMALQAWSSAVPLNFVRVNSGEADIMISFETGDHGDSYPFDGPRGTLAHAFAPGEGLGGDTHFDNAEK
WTMGMNGFNLFTVAAHEFGHALGLAHSTDPSALMYPTYKYQHPFGFHLPKDDVKGIQALYGPRKTYPGKPTVPNAPPHNPSTPDLCDSSSSFDAVTMLGKELLFF
RDRIFWRRQVHVPAGIRPSTITSSFPQLMSNVDAAYEVAERGTAYFFKGPHYWTTRGFQMQGPPRTIYDFGFPRYVQRIDAAVYLKDAEKTLFFVGDEYYSYDER
KRKMEKDYPKYTEEEFSGVNGQIDAAVELNGYIYFFSGPKAYKYDTEKEDVVSVVKSSSWVGC*

Chiroptera
>Myotis
MKVLPASGLALLLITALNFSSAAPSLFAATPRTSRNNYHLAQAYLDKFYTKKGGHQAGEMVVRGSNSIVKKIKELQAFFGLQVTGKLDRSTMDVIKRPRCGVPDV
ANYRLFPGEPKWKKNTLTYRISKYTSSMTSADVDKAVEMALQAWSSAVPLNFVKINSGEADIMISFETGDHGDSYPFDGPRGTLAHAFAPGEGLGGDTHFDNAEK
WTMGMNGFNLFTVAAHEFAHALGLAHSTDPSALMYPTYKYQHPYGFHLPKDDVKGIQALYGPRKPFPGKPTVPNVPPHSPSNPDLCDSSSSFDAVTMLGKELLLF
KDRIFWRRQVHLMAGIRPSTITSSFPQLMSNVDAAYEVAERGTAYFFKGPHYWITRGFHIQGPPRTIYDFGFPRYVQRIDAAVYLKDAQKTLFFVGDEYYSYDER
KRKMEKDYPKNTEEEFSGVNGQIDAAVELNGYIYFFSGPKAYKYDTEKEDVVSVLKSSSWIGC*
>Pteropus
MKVLPASGLALLLVTALNFSTAAPSLFAATPRTSRNNYHLLQAYLVKYYTKKGGHQIGEVVARGSNSMVKKIKELQAFFGLRVTGKLDQSTMDVIKRPRCGVPDV
ANYRLFPGEPKWKKNILTYRISKYTSSMTSADVDKAIEMALRAWSSAVPLNFVRINSGEADIMISFETGDHGDSYPFDGPRGTLAHAFAPGEGLGGDTHFDNAEK
WTMGKNGFNLFTVAAHEFGHALGLAHSTDSSALMYPTYKYQNPYGFRLPKDDVKGIQALYGPRKTFPGKPTVPHTPPHSPSIPDLCDSSSSFDAVTMLGKELLFF
KDRIFWRRQVHLMAGIRPSTITSSFPQLMSNVDAAYEVAERGTAYFFKGPHYWITRGFQIQGPPRTIYEFGFPRYVQRIDAAVYLRDTQKTLFFVGDEYYSYDER
KRKMEKDYPKNTEEEFSGVNGQIDAAVELNGYIYFFSGPKTYKYDTEKEDVVSVLKSSSWIGC*
>Eidolon
MKVLPASGLALLLITALNFSTAAPSLFAATPRTSRNNYHLLQAYLDKYYTKKGGHQIGEVVARGSNSMVKKIKELQAFFGLQVTGKLDQSTMDMIKRPRCGVPDV
ANYRLFPGEPKWKKNTLTYRISKYTPSMTSADVDKAIEMALQAWSSAVPLNFVRINSGEADIMISFETGDHGDSYPFDGPRGTLAHAFAPGEGLGGDTHFDNAEK
WTMGMNGFNLFTVAAHEFGHALGLAHSTDSSALMYPTYKYQHPYGFHLPKDDVKGIQALYGPRKAFPGKPTVPHAPPHSPSIPDLCDSSSSFDAVTMLGKELLFF
KDRIFWRRQVHLMAGIRPSTITSSFPQLMSNVDAAYEVAERGTAYFFKGPHYWITRGFQIQGPPRPIYDFGFPRYVQRIDAAVYLRDAQKTLFFVGDEYYSYDER
KRKMEKDYPKNTEEEFSGVNGQVDAAVELNGYIYFFSGPKTYKYDTEKEDVVSVLKSSSWIGC*
>Eptesicus
MKVLPASGLALLLITALNFSSAAPSLFAATPRTSRNNYHLAQAYLDKYYTKKGGHQAGEMVVRGSNSIVKKIKELQAFFGLQVTGKLDRTTMDVIKRPRCGVPDV
ANYRLFPGEPKWKKNTLTYRISKYTSSMTSADVDKAVEMALQAWSSAVPLNFVKINSGEADIMISFETGDHGDSYPFDGPRGTLAHAFAPGEGLGGDTHFDNAEK
WTMGMNGFNLFTVAAHEFGHALGLAHSTDPSALMYPTYKYQHPYGFHLPKDDVKGIQALYGPRKSFPGKPTVPNVPPHNPSNPDLCDSSSSFDAVTMLGKELLLF
RDRIFWRRQVHLMAGIRPSTITSSFPQLMSNVDAAYEVAERGTAYFFKGPHYWITRGFQIQGPPRTIYDFGFPRYVQRIDAAVYLKDAQKTLFFVGDEYYSYDER
KRKMEKDYPKNTEEEFSGVNGQIDAAVELNGYIYFFSGPKAYKYDTEKEDVVSVLKTSSWIGC*

>Rhinolophus
MKVLPASGLALLLVTALNFSTAAPSLFAATPRTSRNNYHLIQAYLDKYYTKKGGHQVGEIVSRGSNSMVKKIKELQAFFGLQVTGKLDRSTMDVIKRPRCGVPDV
ANYRLFPGEPKWKKNTLTYRVSKYTSSMPSADVDKAIEMALQAWSSAVPLNFVRINSGEADIMISFETGDHGDSYPFDGPRGTLAHAFAPGEGLGGDTHFDNAEK
WTMGTNGFNLFTVAAHEFGHALGLAHSTDPTALMYPTYKYQHPYGFHLPKDDVKGIQALYGPRKTFPGKPTVPHVPPHTPSNPDLCDSSSSFDAVTMLGKELLFF
KDRIFWRRQVHPMAGIRPSTITSSFPQLMSNVDAAYEVAERGTAYFFKGPHYWITRGFQMQGPPRTIYDFGFPRYVQRIDAAVYLKDAQKTLFFVGDEYYSYDER
KRKMEKDYPKNTEEEFSGVNGQIDAAVELNGYIYFFSGPKAYKYDTEKEDVVSVLKSSSWIGC*
>Pteronotus
MKVLPASGLVLLLIMALNLSTAAPSLFAATPRTSRNNYHIAQAYLDKYYTRKGGHQVGEMAARGSNSIVKKIKELQAFFGLQVTGKLDRSTMDVIKRPRCGVPDV
ANYRLFPGEPKWKKNTLTYRISKYTSSMTSADVDKAVEMALQAWSSAVPLNFVRINSGEADIMISFETGDHGDSYPFDGPRGTLAHAFAPGEGLGGDTHFDNAEK
WTMGTNGFNLFTVAAHEFGHALGLAHSTDPSALMYPTYKYQHPYGFHLPKDDVKGIQALYGPRKTFPGKPTVPDVPPHNPSIPDLCDSSSSFDAVTMLGKELLLF
RDRIFWRRQVHLMAGIRPSTITSSFPQLMSNVDAAYEVAERGTAYFFKGPHYWITRGFQMQGPPRTIYDFGFPRYVQRIDAAVYLKDAQKTLFFVGDEYYSYDER
KMKMEKDYPKNTEEEFSGVNGQIDAAVELNGYIYFFSGPKTYKYDIEKEDVVSVLKSSSWIGC*
>Megaderma
MKVLPASVLALLLIAALNLPTAAPSLFAATLRTSRTNFHLAQAYLDKYYTKKGGHQAAEMVARGSNSMVKKIKELQAFFGLQITGKLDRMTMDVIKRPRCGVPDV
ANYRLFPGEPKWKKHTLTYRVSKYTSSMTSADVDKAVEMALQAWSSAVPLNFVRINSGEADIMISFETGDHGDSYPFDGPRGTLAHAFAPGEGLGGDTHFDNAEK
WTMGMNGFNLFTVAAHEFGHALGLAHSTDPSALMYPTYKYQHPYGFHLPKDDVKGIQALYGPRKTFPGKPTVPHSPPHQPSDPDPCDSSSSFDAVTMLGKELLFF
KDRIFWRRQVHLMAGIRPSTITSSFPQLMSNVDAAYEVAERGTAYFFKGPHYWITRGFQMQGPPRTIYDFGFPRYVQRIDAAVYLRDPQKTLFFVADEYYSYDER
KRKMEKDYPKNTEEEFSGVNGQIDAAVELNGYIYFFSGPKTYKYDTEKEDVVSVLKSSSWIGC*

Afrotheria
>Loxodonta
MKVLPASGLAVLFITTLKFSTAAPSLFAATSRTSRNNYQLAQAYLDKYYTKEGGHQIGEMVARGGNAMVKKIKELQAFFGLKVTGKLDQLTIDVIKKPRCGVPDV
ANYRLFPGEPKWKKNTLTYRISKYTSSMSSADVDKAIEMALQAWSSAIPLSFVKLNTGEADIMISFETGDHGDSYPFDGPRGTLAHAFAPGEGLGGDTHFDNAEK
WTMGMNGFNLFTVAAHEFGHALGLAHSTDPSALMYPTYKYQHPYGFRLPKDDVKGIQALYGPRKTFPGKPTVPHGPPQNPSTPDLCDSSSSFDAVTMLGKELLFF
KDRIFWRRQAHLMAGIRPSTITSSFPQIMANVDAAYEVAERGAAYFFKGPHYWVTRGFQMQGPPRSIYDFGFPRFVQQIDAAVYLKNAQKTLFFVGDEYYSYDER
KGKMEKDYPKNTEEEFSGVSGQIDAAVELNGYIYFFSGPKAYKYDTEKEDVVSVLKSSSWIGC*
>Procavia
MKVLPASGLAVLLITTLKFSTAAPSLFAATFGNSRNNYHLAQAYLDKYYTEKGGHQVGEMVAKGGNSMVKKIKELQAFFGLKVTGKLDQLTMNVIKKPRCGVPDV
ANYRLFPGEPKWKKNTLTYR???DHGDSYPFDGPRGTLAHAFAPGEGLGGDTHFDNAEK
WTMGMNGFNLFTVAAHEFGHALGLAHSTDPSALMYPTYKYQHPYGFRLPRDDVKGIQALYGPRKTPPGKPTLPHAPPHNPSIPDLCDSSSSFDAVTMLGKELLFF
KDRIFWRRQVHLAAGIRPSTITSSFPQLMSNVDAAYEVAERGTAYFFKGPHYWVTRGFQMQGPPRSIYDFGFPRFVQQIDAAVYLKDVQKTLFFVGDEYYR????
???????????????????IG?IDAAVELNGYIYFFSGPKAYKYDTEKEDVVSVIKANSWIGC*
>Elephantulus
MKVLLASGFTVLLISALKFSTAAPSLFAATSRTSRNNYHLAQEYLDKYYTEKGGQQMGEMVARRGNSMVKKIKELQAFFGLQITGKLDQKTMDVIKKPRCGVPDV
ANYRLFPGEPKWKKNILTYRISKYTSSMSPSEVDKAVEMALQAWSSAIPLNFVKIHSGEADIMISFETGDHGDSYPFDGPRGTLAHAFAPGEGLGGDTHFDNAEK
WTMGMNGFNLFTVAAHEFGHALGLAHSTDPSALMYPTYKYQHPFGFRLPKDDVRGIQALYIFWRRQVHLTAGIRPSTITSSFPQLMSNVDAAYEVAERGVAYFFK
GPHYWVTRGFQMQGPPRTIYDFGFPRYVQRIDAAVYLKEPQKTLFFVGDEYYSFDERKMKMEKDYPKNTEEEFSGVIGQIDAAVELNGYVYFFSGPKTYKYDMEK
EDVVSVVKSSSWIGC*
>Echinops
MKVLPASGLVVLLITALKCSTAAPSLFAATSRTSRNNYHLAQAYLEKFYPKKGEHQVGEMVARGGNSMIRKIKELQAFFGLQVTGKLDQQTMNVIKKPRCGVPDV
ANYRLFPGEPKWKKNTLTYRISKYTSSMSSAEVDKAVEMALQAWSSAIPLNFVKVNLGEADIMISFETGDHGDSYPFDGPRGTLAHAFAPGEGLGGDTHFDNAEK
WTMGMNGFNLFTVAAHEFGHALGLAHSTDPSALMYPTYKYQHPYGFHLPKDDVKGIQALYGPRKTLPGKPTMPHLPPKKPSNPDLCDSNSPFDAVTMLGKELLFF
RDRIFWRRQVHLTTGIRPSTITSSFPQLMSNVDAAYEVAERGAAYFFKGPHYWVTRGFQMQGPPRTIYDFGFPRYVQRLDAAVYLKEAQKTLFFVGNEYYSYDER
KRKMEKDYPKNIEEEFSGVNGQIDAAVELNGYIYFFSGPKAYKYDTEKEDVVSVVKSSSWIGC*
>Chrysochloris
MKVLPVSGLAVLLITALKFSTAAPSLFTATSRSSRNNYHLAQAYLDKYYTKKGGHQVGEMVARGGNPMVRKIKELQAFFGLQVTGKLDQSTMNVIKRPRCGVPDV
ANYRLFPGEPKWKKNTLTYRISKYTSSMSSADVDKAVEMALQAWSSAIPLNFVKVTSGEADIMISFETGDHGDSYPFDGPRGTLAHAFAPGEGLGGDTHFDNAEK
WTMGMNGFNLFTVAAHEFGHALGLAHSTDPSALMYPTYKYQHPYGFHLPKDDVKGIQALYGPRKTLSGKPTVPHAPPQTPSNPDLCDSSSSFDAVTMLGKELLFF
RDRVFWRRQVHLTAGIQPSTITSSFPQLMSNVDAAYEVAERGIAYFFKGPHYWVTRGFQMQGPPRTIYDFGFPRFVQRIDAAVYLKEAQKTLFFVGDEYYSFDER
KGKMEKDYPKNTEEEFSGVNGQIDAAVELNGYIYFFSGPKAYKYDIEKEDVVSVIKSSSWIGC*
>Trichechus
MKVLPASGLAVLFITTLKFSTAAPSLFAATSRTSRTNYHLAQAYLDKYYTNKGGHRIGEMVARGGNSMVKKIKELQAFFGLKVTGKLDQRTMNVIKKPRCGVPDV
ANYRLFPGEPKWKKNTLTYRIAKYTPSMSSADVDKAVEMALRAWSSAIPLSFVKLNSGEADIMISFETGDHGDSYPFDGPRGTLAHAFAPGEGLGGDTHFDNAEK
WTMGMNGFNLFTVAAHEFGHALGLAHSTDPSALMYPTYKYKHPYGFRLPKDDVKGIQALYGPRKTFPGKPTVPHAPAHNPSIPDLCDSSSSFDAVTMLGKELLFF
KDRIFWRRHVHFMAGIQPSTITSSFPQLMSNVDAAYEVAERGTAYFFKGPHYWVTRGFQMQGPPRSIYDFGFPKFVQQIDAAVYLKDAQKTLFFVGDEYYSYDER
KRKMEKDYPKNTEEEFSGVSGQIDAAVELNGYIYFFSGPKAYKYDTEKEDVVSVVKSSSWIGC*

Marsupiala
>Sarcophilus
MKILQASGLSFLLITALKLSAIASTLFVSPARSTRRDYYIAQAYLDRYYTKKGGHQIGEIVVKGRNTMERKIKEMQAFFGLQVTGKLDYSTMKVMKRPRCGVPDI
ANYRLFPGEPKWKKNTLTYRVSKYTSSMSHAEVDKAVDMALQAWSNAVPLNFVRQNTGEADIMISFELGDHGDSYPFDGPRGTLAHAFAPGEGLGGDTHFDNAEK
WTMGTNGFNLFTVAAHEFGHALGLAHSSDPSALMYPTYRYQHPYGFRLPKDDVKGIQALYGPRKAVTERPNVPHIPPHKPSDPDICDSTSSFDAVTMLGKELLFF
KDRIFWRRQVHLAAGIRPSTITSSFPQLMSNVDAAYEVTEKGIAFFFKGPHYWITRGFQMQGPPRTIYDFGFPRHVQQIDAAVYLREPRKTLFFVGEDYYSYDEV
KRKMEKDYPKNIEEEFSGITGKIDAAVEVNGYLYFFSGPKAYKYDIEKEDVVNVVKSSSWIGC*
>Monodelphis
MKTLQASGLYFLLITTLKLSAIASALFASPPRSTRRDYYIAQAYLDRYYTKKGGHQIGEMVVKGRNTMERKIKEMQAFFGLRVTGKLDYSTMKVIKRPRCGVPDI
ANYRLFPGEPKWKKNTLTYRVSKYTSSMTHAEVDKAVDMALQAWSNAVPLNFVRQNTGEADIMISFEIGDHGDSYPFDGPRGTLAHAFAPGEGLGGDTHFDNAEK
WTMGTNGFNLFTVAAHEFGHALGLAHSTDPSALMYPTYKYQHPYGFRLPKDDVKGIQALYGPRKTATERPNVPNVPPHNPSDPDVCDSSASFDAVTMLGKELLFF
KDRIFWRRQVHLAAGIRPSTITSSFPQLMSNVDAAYEVTERGIAFFFKGPHYWITRGFQMQGPPRTIYDFGFPRHVQQIDAAVYLREPRKTLFFVGEDYYSFDEV
KRKMEKDYPKNVEEEFSGIIGKIDAAVEMNGYIYFFSGPKSYKYDIEKEDVVNVMKSSAWIGC*
>Macropus
MKILQASGLSFILITALKLSAVASALFASPARSTRKDYYIAQAYLDRYYTKKGGHHIGEMVVKGRNNMERKIKEMQAFFGLQVTGKLDYSTMKMMKKPRCGVPDI
ANYRLFPGEPKWKKNILTYRVSKYTSSMSHAEVDKAVDMALQAWSNAVPLNFVRLDTGEADIMISFEIGDHGDSYPFDGPRGTLAHAFAPGEGLGGDTHFDNAEK
WTMGMNGFNLFTVAAHEFGHALGLAHSSDPTALMYPTYKYQHPYGFRLPRDDVKGIQALYGPRKPVTERPNVPNVPPHKPSDPDVCDSTSSFDAVTMLGKELLFF
KDRLFWRRQAHMSAGIRPSTITSSFPQLMSNVDAAYEVPERGIAFFFKGPHYWMTRGFKMQGPPRTIYDFGFPRHVQHIDAAVYLREPRKTLFFVGEDYYSYDEV
KRKMEKDYPKNIEEEFSGIIGNIDAAVEVNGYVYFFSGPKAYKYDIEKEDVVNVVRSSSWIGC*
