# Supplementary material for: Evolutionary Analysis Predicts Sensitive Positions of MMP20 and Validates Newly- and Previously-Identified MMP20 Mutations Causing Amelogenesis Imperfecta
Source: Front Physiol. 2017 Jun 14;8:398. doi: 10.3389/fphys.2017.00398 (PMC5469888; doi:10.3389/fphys.2017.00398)
Supplement: Supplementary file 4 [file DataSheet2.PDF]

A.

MMP20 normal sequence

Splice donor sites, direct strand

pos 5'->3'	phase	strand	confidence	5' exon intron 3'
**140**	**0**	**+**	**0.36**	**CCTCGCACAG^GTTTGTGAAT**
597	1	+	0.35	TATATTCAAG^GTAACCCTGA
1665	0	+	0.34	AATGGCAGAG^GTTAGCCTAG
2694	1	+	0.46	AAAGTGATCA^GTGAGCCTGG
2973	0	+	0.54	CCAAGGACAG^GTGAGGCCAG
3256	0	+	0.46	TTTGAAAATG^GTAAGATGGC
4028	0	+	0.65	ATAGGCCAAG^GTAAACATCC
4219	2	+	0.34	AGAGCCAAAG^GTATCCATCT

MMP20 mutated sequence

Splice donor sites, direct strand

pos 5'->3'	phase	strand	confidence	5' exon intron 3'
597	1	+	0.35	TATATTCAAG^GTAACCCTGA
1665	0	+	0.34	AATGGCAGAG^GTTAGCCTAG
2694	1	+	0.46	AAAGTGATCA^GTGAGCCTGG
2973	0	+	0.54	CCAAGGACAG^GTGAGGCCAG
3256	0	+	0.46	TTTGAAAATG^GTAAGATGGC
4028	0	+	0.65	ATAGGCCAAG^GTAAACATCC
4219	2	+	0.34	AGAGCCAAAG^GTATCCATCT

B.

```
>MMP20, normal splice site
AGGTTTGTG   MAXENT: -16.62   MDD: -7.23  MM: -9.24   WMM: -10.24

>MMP20 mutated splice site
AGGTTTGGG   MAXENT: -9.53    MDD: -6.23  MM: -5.75   WMM: -6.33
```

Supplementary Data 2. Analyses of the mutated splice donor site using NetGen2 (A) and MaxEntScan (B).

A. NetGen2 analysis of the first 5,000 bp of the normal and mutated human *MMP20* sequences. NetGen2 detects all potential splice donor sites. Position 140 corresponds to the normal splice donor site in intron1. This splice site is not detected in the mutated sequence.

B. MaxEntScan analysis of the normal and mutated splice donor site in intron1 of human *MMP20*. MaxEntScan gives the splice score using three different models: MAXENT= Maximum Entropy Model; MM= Markov Model ; WMM = Weight Matrix Model. The mutated MMP20 sequence shows a reduced score for the three models meaning a reduced probability of splicing.
