# Supplementary material for: Evolutionary Analysis Predicts Sensitive Positions of MMP20 and Validates Newly- and Previously-Identified MMP20 Mutations Causing Amelogenesis Imperfecta
Source: Front Physiol. 2017 Jun 14;8:398. doi: 10.3389/fphys.2017.00398 (PMC5469888; doi:10.3389/fphys.2017.00398)
Supplement: Supplementary file 5 [file Image1.PDF]

Supplementary Figure 1. Alignment of the 75 amino acid sequences of mammalian MMP20 used in the evolutionary analysis. The human sequence was used as a reference sequence in this alignment. Signal peptide underlined. Substituted positions leading to AI are on grey background. (|) : exon limits; (.) : amino acid identical to human MMP20 residue; (-) : amino acid deletion; (?) : unknown amino acid. See supplementary Table 1 for species names and references, and supplementary Data 1 for the amino acid sequences.

```
                 1 Exon 1                          35      | Exon 2                                  84
Homo             MKVLPASGLAVFLIMALKFSTAAPSLVAASPRTWRNNYRLAQ AYLDKYYTNKEGHQIGEMVARGSNSMIRKIKELQAFFGLQVT
Pan              .......................................... ..........................................
Gorilla          .......................................... ..........................................
Pongo            ...........L.............................. ......................G...................
Nomascus         ...........L.............................. ....T.........T.......G...................
Papio            ...........L.............................. ......................G...V............R..
Mandrillus       ...........L.............................. ......................G...V............R..
Macaca           ...........L.............................. ......................G...V............R..
Chlorocebus      ...........L.............................. ......................G...V............R..
Rhinopithecus    ...........L.............................. ......................G...V...............
Colobus          ...........L.............................. ......................G...V...............
Callithrix       ...........L.M...................L........ ..V................S..G..LV...............
Saimiri          ...........L....................I......... ..V................S..G...V...............
Aotus            ...........L.............................. ..V................S..G...V...............
Tarsius          ...........L..T...L.......F..T........H... ........R.G...V...A...G...V............R..
Otolemur         ..M........L..T...L.......FT.T........H... E.......K.G...........G...VK..............
Daubentonia      ..M........L..T...L.......FS.A........H... ........K.GE.....V....G...VK..............
Microcebus       ...........L..T...L.......FS.T........H... ........K.G....A..A...G...VK..............
Galeopterus      .R......I..L.VT.....S.....F..T...L....H... ........K.G.........K.GS..V...............
Tupaia           ...........L..T.....S.V...F.GT..SL..D.H... ........R.G..P........G...VK..............
Mus              ...........L-VT....A..D.N.L..T...F.S..H... ........K.G.P.A......E..P...R.....I....K..
Rattus           ...........L-VT....A....A.L..T...F.S..H... ........K.G.P.A......E..PTV.......T....R..
Cricetulus       ..G........L-VT....A....T.F..A...F.R..HI.. E......SQ.G.L.A......E..P.........S.......
Mesocricetus     ...........L-VA....A....T.F..A...S.SS.HI.. E......SQ.G.L.A......ED.P.........S.......
Microtus         ...........L-VT....A..V.T.F..T...F.SS.Q... D.......P.G.P.A....V.QG.P.V.......S.......
Jaculus          ..A........L.VT.....A...T.F..T........H... .....F..K.G...V..LAV..G...VK......T.......
Dipodomys        ...........L.VT...L...F...FV.T........H... ...Q....S.GNL.M.......GSPLVK.V......L.....
Cavia            ...........L.LT.....S...T.F..T.......FH... ........K.G...V....S..G...VK......T....PL.
Octodon          ...........L.VT..E..S.T...F..T.......FH... ...E...MKRG...A....S.S.MA--.R..D....L..R..
Chinchilla       ..A........L.VT.....S...-.L..T.......FH... ........KRG...V....S..G...VK........L.....
Fukomys          ..M........L.VT.....ST..T.F..T.......FH... ..I.....K.D...V....S..G.P.AK..............
Heterocephalus   ...........L.VT.....S.D.TVF..T.......FH... ........K.GSL.V....S..G.P.VK..............
Nannospalax      ...........L.VT....A....T.F..TL.SS...HH... ........KNGV..A.......G...VK......T.......
Ictidomys        ...........L..T...........F..T........H... E.......K.G..PV...A...G.A.VK.............N
Oryctolagus      ...........L.VT.......T...F..T.......FH... ........R.G.S.........G...VK...........R..
Ochotona         ....................A.....FT.T.......FH... ........K.G.T.V.......G.A.VK..............
Bos              ..M........L.VT...........P......S........ ........K.G.P.........G..TVK......E....R..
Capra            ...........L..T...........L......S........ ........R.G.P.........G..TVK......E....R..
Ovis             ...........L..T...........L......S........ ........R.G.P.........G..TVK......E....R..
Pantholops       ...........L..T...........L......S........ E.......K.G.P.........G..TVK......E....R..
Tursiops         ...F.......L.VT...........F..T...S..S.H... ........K.G.......I...G...VKN..........R..
Orcinus          ...........L.VT...........F..T...S..S.H... ........K.G.......I...G...VKN..........R..
Lipotes          ...........L.VT...........F..T...S..S.H... ........K.G..R....I...G...VKN..........R..
Physeter         ...I.......L..T...........F..T...S..S.H... ........K.G.......I...G...VKN..........R..
Vicugna          ...........L.VT.....S.....F..T...S....H... ........K.G..PL...A.......VK......Q....R..
Camelus          ...........L.VT.....S.....F..T...S....H... ........K.G..PL...A...G...VK......Q....R..
Sus              ...........L.VT.....A.....F..T...S....H... ........K.G...V.....K.G...VK...........R..
Equus            ....L......L..T.......T...F..T...S....H... ........V.G...............VK......V....P..
Ceratotherium    ...FL......L..T.......T...F..T...S....H... ........V.G...V........K..VK......V....P..
Canis            .T...MC...LL.GA..E.C.....VS..A...TQ.K.H... ........S.A.P.V...G.P.GRAL.K...........RI.
Ailuropoda       ...........L..A...........L.DT.G.A...FH... ........R.A.P.V..LG.P.GRALVK...........R..
Ursus            ...........L..A...........F..T.G.A...FH... ........R.A.P.V...G.P.GRALVK...........R..
Leptonychotes    ..........LL.TT...........F..T...S........ ........R.A.P.V...G.P.GRALVK...........R..
Odobenus         .....T....LL..T...........F..T...S....H... ........R.A.P.V...G.P.GRALVK...........R..
Mustela          ......P...LL.LT..EL.AT....S..T.G.S....H... ........REA.P.V...G.A.GRALVK...........R..
Felis            ...........L.VT..........PP......S....H... ........R.G...L...A...AS.LVK...........R..
Erinaceus        .....T..F..L......L.......F..T...S....H... ....R...K..EY..........H...K............I.
Sorex            ........F..L.FAT.......S..L..A...SW...H... ...N....KEG......L...RG...VK......T..N....
Condylura        ...........L..T...L.......F..TH..S....H... ........K.G.Q.............VK............I.
Myotis           ..........LL..T..N..S.....F..T...S....H... .....F..K.G...A....V.....IVK..............
Pteropus         ..........LL.VT..N........F..T...S....H.L. ...V....K.G......V........VK...........R..
Eidolon          ..........LL..T..N........F..T...S....H.L. ........K.G......V........VK..............
Eptesicus        ..........LL..T..N..S.....F..T...S....H... ........K.G...A....V.....IVK..............
Rhinolophus      ..........LL.VT..N........F..T...S....H.I. ........K.G...V.I.S.......VK..............
Pteronotus       .........VLL.....NL.......F..T...S....HI.. ........R.G...V...A......IVK..............
Megaderma        .......V..LL..A..NLP......F..TL..S.T.FH... ........K.G...AA..........VK............I.
Loxodonta        ...........LF.TT..........F..TS..S....Q... ........KEG...........G.A.VK...........K..
Procavia         ...........L..TT..........F..TFGNS....H... ........E.G...V.....K.G...VK...........K..
Elephantulus     ....L...FT.L..S...........F..TS..S....H... E.......E.G.Q.M......RG...VK............I.
Echinops         .........V.L..T...C.......F..TS..S....H... ...E.F.PK.GE..V.......G...................
Chrysochloris    .....V.....L..T...........FT.TS.SS....H... ........K.G...V.......G.P.V...............
Trichechus       ...........LF.TT..........F..TS..S.T..H... ..........G..R........G...VK...........K..
Sarcophilus      ..I.Q....SFL..T...L.AI.ST.FVSPA.ST.RD.YI.. ....R...K.G......I.VK.R.T.E.....M.........
Monodelphis      ..T.Q....YFL..TT..L.AI.SA.F.SP..ST.RD.YI.. ....R...K.G........VK.R.T.E.....M......R..
Macropus         ..I.Q....SFI..T...L.AV.SA.F.SPA.ST.KD.YI.. ....R...K.G..H.....VK.R.N.E.....M.........
```

```
                85                     108               |   130      Exon 3                      168
Homo            GKLDQTTMNVIKKPRCGVPDVANYRLFPGEPKWKKNTLTYR ISKYTPSMSSVEVDKAVEMALQAWSSAVPLSFVRINSGEADIM
Pan             ....................................I.... ...........................................
Gorilla         ......................................... ...........................................
Pongo           ....K.................................... ...........................................
Nomascus        ......................................... .........P.................A...............
Papio           ........D...........................I.... V....T....A..E...Q.........................
Mandrillus      ........D...........................I.... V....T....A..E...Q.........................
Macaca          ........D...........................I.... V....T....A..E...Q.........................
Chlorocebus     ........D...........................I.... V....T....A..E...Q.........................
Rhinopithecus   ....P...D...........................I.... .....T....A..E...Q.........I..N............
Colobus         ........D...........................I.... .....T....A..E...Q.........I..N............
Callithrix      ....K...D................................ .........PA......G.......G....N............
Saimiri         ....K...D..........................T..... .........PA.....MG............N............
Aotus           ....K...D................................ .........PA......G............N............
Tarsius         ....P...D................................ .....S...AA..............G........V........
Otolemur        ....A...D...R............................ ..........A...T.I....R........N...V.T......
Daubentonia     ....R...D................................ .........AA....SI....R........N...V.A......
Microcebus      ....P...D...R............................ .........AA.....ID...R........N...V.A......
Galeopterus     ........D.M.R............................ .........A...............G....N...VLA......
Tupaia          ........D...R............................ ..........AD......................V........
Mus             .....N..............................I.... .........PT.....IQ...H...T....N............
Rattus          .....N..D...R.......................I.... V........PT......H...H...T....I............
Cricetulus      .....N......R.......................I.... V....S...PA..........R...T....N............
Mesocricetus    .....N......R.......................I.... V........PA..........R...T....N............
Microtus        ....P...H...R.......................I.... V........PA..........R...T....N............
Jaculus         .....N..D...R.......................M.... ........RPA..........R........N...THA......
Dipodomys       ....RN..DM............................... ........RPA..........R........N............
Cavia           .....S..D........................E....... ....AA...PA..ER.I....R........N............
Octodon         .....R..DT..Q....................E....... VA..AA..RPA...R.I....R........N....H.......
Chinchilla      .....N..D...M.......M............E....... ....AA..RPA...R.I....R........N...V........
Fukomys         .....N..D........................E..I.... ....AS...PA...R.I.............N..K.S.......
Heterocephalus  .....N..D........................E....... ....AS...PA...R.IQ............N............
Nannospalax     .....N..D...R.......................I.... V....S...PP.....I....R...T......I.V........
Ictidomys       .....N..D...R............................ .A...S..RPI..E.............................
Oryctolagus     .....N..D................................ VA...S..TPF.....I....R............V.A......
Ochotona        .....N..D................................ V....S..AP...........R........N...V.T......
Bos             ....RA..D...R............................ ........TPA...R.M....R........N.....A......
Capra           .....A..D...R.........D.................. ........TPA...R.M....R........N...V.A......
Ovis            ....RA..D...R.........D.................. ........TPA...R.M....R........N...V.A......
Pantholops      ....RA.LD...R.........D.................. ........TPA...R.M....R........N...V.A......
Tursiops        ....RA..D...R............................ ........T.A................................
Orcinus         ....RA..D...R............................ ........T.A................................
Lipotes         ....RA..D...R.......................I.... ........T.A................I...............
Physeter        ....RA..D...R.......................P.... ........T.A...................N............
Vicugna         ....RA..D...R............................ ........TPA...R..Q................V.A......
Camelus         ....RA..D...R............................ ........TPA...R..Q................V.A......
Sus             ....R...D...R............................ ........TPA.....M.................V.A......
Equus           ....KS..D...R............................ .....T..TPAD.N................N...VD.......
Ceratotherium   ....KP..D...R............................ .....T..T.AD.....Q................VD.......
Canis           ....RP..DM..R............................ .....S...PA.............G.......I.V........
Ailuropoda      ....RR..D...R............................ ........TPA...................G...V.A......
Ursus           ....RR..D...R............................ ........APA...................G...V.A......
Leptonychotes   .R..RP..D...R............................ ........T.A...R.......T.........A.V.A......
Odobenus        ....RP..D...R............................ ........T.A.......................V.T......
Mustela         ....RP..D...R.......................S.... ........P.A.......................V.A......
Felis           ....RS..D...R............................ ........AAAD............G.....T...VT.......
Erinaceus       ....R...D................................ .....S..T.A...................N..K.........
Sorex           ....KS..D...R............................ .....S..TPA.......................V........
Condylura       ....RS..DM............................... .....S..T.A...................N...V........
Myotis          ....RS..D...R............................ .....S..T.AD..................N..K.........
Pteropus        .....S..D...R.......................I.... .....S..T.AD....I....R........N............
Eidolon         .....S..DM..R............................ ........T.AD....I.............N............
Eptesicus       ....R...D...R............................ .....S..T.AD..................N..K.........
Rhinolophus     ....RS..D...R............................ V....S..P.AD....I.............N............
Pteronotus      ....RS..D...R............................ .....S..T.AD..................N............
Megaderma       ....RM..D...R......................H..... V....S..T.AD..................N............
Loxodonta       .....L.ID................................ .....S....AD....I..........I.....KL.T......
Procavia        .....L................................... ???????????????????????????????????????????
Elephantulus    .....K..D...........................I.... .....S...PS................I..N..K.H.......
Echinops        .....Q................................... .....S....A................I..N..KV.L......
Chrysochloris   .....S......R............................ .....S....AD...............I..N..KVT.......
Trichechus      .....R................................... .A........AD.........R.....I.....KL........
Sarcophilus     ....YS..K.M.R.......I.................... V....S...HA......D.......N....N...Q.T......
Monodelphis     ....YS..K...R.......I.................... V....S..THA......D.......N....N...Q.T......
Macropus        ....YS..KMM.........I...............I.... V....S...HA......D.......N....N...LDT......
```

```
               169    | Exon 4      189            204           | Exon 5 226                     251
Homo            ISFENG DHGDSYPFDGPRGTLAHAFAPGEGLGGDTHFDNAEKWTMGTN GFNLFTVAAHEFGHALGLAHSTDPSALMYPTYKYK
Pan             ...... .......................................... ...................................
Gorilla         ...... .......................................... ...................................
Pongo           ...... .......................................... ..................................Q
Nomascus        ...... .......................................... ...................................
Papio           ...... .......................................... ..................................Q
Mandrillus      ...... .......................................... ..................................Q
Macaca          ...... .......................................... ..................................Q
Chlorocebus     ...... .......................................... ..................................Q
Rhinopithecus   ...... ........................................K. ..................................Q
Colobus         ...... .......................................... ..................................Q
Callithrix      V...T. .......................................... ................................R.Q
Saimiri         ....T. .......................................... ..................................Q
Aotus           ....T. .......................................... ..................................Q
Tarsius         ....T. ........................................M. .....................S............Q
Otolemur        ....T. ......................................L.M. ..................................Q
Daubentonia     ....T. ........................................M. ......................N...........Q
Microcebus      ....T. ........................................M. ..................................Q
Galeopterus     ....T. ........................................K. ..................................Q
Tupaia          ....T. ........................................M. ........................T.........Q
Mus             ....T. .......................................... ..................G...............Q
Rattus          ....T. .......................................... ..................G...............Q
Cricetulus      ....T. ........................................M. ..................G...............Q
Mesocricetus    ....T. ........................................M. ..................G...............Q
Microtus        ....T. ........................................M. ..................G...............Q
Jaculus         ....S. .......................................... ..................................Q
Dipodomys       ....T. .......................................... ..................................Q
Cavia           V...T. ......................................L.M. ...................................
Octodon         V...T. ......................................L.M. ...................................
Chinchilla      V...T. ......................................L.M. ...................................
Fukomys         V...T. ......................................L.M. ...................................
Heterocephalus  V...T. ......................................L.M. ...................................
Nannospalax     ....T. ........................................M. ..................................Q
Ictidomys       ....T. ........................................M. .....................S..T.........Q
Oryctolagus     ....T. .......................................... ..................................Q
Ochotona        ....T. .......................................... ........................T.........Q
Bos             ....T. .......................................... ..................................Q
Capra           ....T. .......................................... ..................................Q
Ovis            ....T. .......................................... ..................................Q
Pantholops      ....T. .......................................... ..................................Q
Tursiops        ....T. ......................G.................M. ..................................Q
Orcinus         ....T. ........................................M. ..................................Q
Lipotes         ....T. ........................................M. ..............................A...Q
Physeter        ....T. ........................................M. ..................................Q
Vicugna         ....T. .......................................... ..................................Q
Camelus         ....T. .......................................... ...................................
Sus             ....T. ........................................M. ..................................Q
Equus           ....T. .......................................... ..................................Q
Ceratotherium   ....T. ........................................M. ..................................Q
Canis           ....T. ........................................M. ..................................Q
Ailuropoda      ....T. ........................................M. ..................................Q
Ursus           ....T. ........................................M. ..................................Q
Leptonychotes   ....T. ........................................M. ..................................Q
Odobenus        ....T. .......................................... ..................................Q
Mustela         ....T. ........................................M. ..................................Q
Felis           ....S. ........................................M. ..................................Q
Erinaceus       ....T. ........................................M. ..................................Q
Sorex           ....T. .......................................... .....................S............Q
Condylura       ....T. ........................................M. ..................................Q
Myotis          ....T. ........................................M. ............A.....................Q
Pteropus        ....T. ........................................K. .......................S..........Q
Eidolon         ....T. ........................................M. .......................S..........Q
Eptesicus       ....T. ........................................M. ..................................Q
Rhinolophus     ....T. .......................................... ........................T.........Q
Pteronotus      ....T. .......................................... ..................................Q
Megaderma       ....T. ........................................M. ..................................Q
Loxodonta       ....T. ........................................M. ..................................Q
Procavia        ?????? ........................................M. ..................................Q
Elephantulus    ....T. ........................................M. ..................................Q
Echinops        ....T. ........................................M. ..................................Q
Chrysochloris   ....T. ........................................M. ..................................Q
Trichechus      ....T. ........................................M. ...................................
Sarcophilus     ....L. .......................................... .....................S..........R.Q
Monodelphis     ....I. .......................................... ..................................Q
Macropus        ....I. ........................................M. .....................S..T.........Q
```

```
                 252                 | Exon 6                          304              | Exon 7       334
Homo             NPYGFHLPKDDVKGIQALY GPRKVFLGKPTLPHAPHHKPSIPDLCDSSSSFDAVTMLGKELLLFKDR IFWRRQVHLRTGIRPS
Pan              ................... ....A........................................... ................
Gorilla          ...R............... ....A........................................... ................
Pongo            ................... ....A................T.......................... ................
Nomascus         ................... ....P......V....PY...T.......................... ................
Papio            ................... ....P.....MV....P....T........................G. .....E.P........
Mandrillus       ................... ....P.....MV....P....T........................G. ................
Macaca           ................... ....P.....MV.........T........................G. ................
Chlorocebus      ................... ....P.....MV....A....T........................G. ................
Rhinopithecus    ................... ....P.....MV....P....T........................G. ................
Colobus          ................... ....P.....MV....P....T........................G. ................
Callithrix       ................... ....P......V....P....V..............V..R........ ..........A.....
Saimiri          ................... ....P......V....P....T.......................... ..........A.....
Aotus            ................... ....S...........P....T.......................... ..........A.....
Tarsius          ................... ....P......V..V.P....T.....................F.... .........PA.....
Otolemur         ........M.......... ....P......M..G.P.N.PT.....................F.... .........P......
Daubentonia      ........S.......... ....P...N.SM..G.P...P........A.............F.R.. ......L..PS.....
Microcebus       .....R..S.......... ....PY..N.AM..G.P...PT.......A.............F.... .........PS.....
Galeopterus      D..D............... ....T.....SV.LG.P.R...........Y............F.R.. .........MA.....
Tupaia           ................... ....T......V....P..........................F.... .........TA.....
Mus              ...R............... ....I.P....M..I.P..........................F.... .........P......
Rattus           ...R............... ....T.P....M..I.P..........................F.... .....R...PA.....
Cricetulus       ..SR............... ....T.P....V..I.P....F.....A.................... .........AA....N
Mesocricetus     ...R............... ....T.P....V..I.P....F.....T..................G. .........AA....N
Microtus         ...R............... ....T.P....M..I.P....L.......................... .........AA....N
Jaculus          H.F..R............. ....T.P....M..V.S..........................F.... .........PS.....
Dipodomys        Q....R.......A..P.. .L..P......A..I.P..........................F.... .........PS.....
Cavia            .....R............. ....PY.....V..L.PR......V...................F.... .........LSE...R
Octodon          .....R............. ....P.....MG..L.P.......V....................... .........LAE..A.
Chinchilla       .....R............. ....P......I..I.P....F..V..A.................... .........LAE....
Fukomys          .....R............. ....P...N.....G.P.......I...................F.... .........LAE....
Heterocephalus   .....R............. ....P......I..V.P.......V...L...............F.... .........LAE....
Nannospalax      ..FR............... ....T.P....V..V.P.T....................N...F.... ........VPA.....
Ictidomys        .....R............. ....P......I..V.P....N..P...RA.............F.R.. ........VPA.....
Oryctolagus      ..F..R............. ....P.....HM..I.P..........................F.R.. .V....A..P..VQ..
Ochotona         ............R...... ....P.........I.P...IL.....................F.R.. ......L..P..VQ..
Bos              .....R............. ...RA.S....A..G.P.N.........NL...............R.. .........MS.....
Capra            ................... ...RA.P....A..G.P.N.........NL...............R.. .........MS.....
Ovis             ................... ...RA.P....A..G.P.N.........NL...............R.. .........MS.....
Pantholops       ................... ...RA.P....A..G.P.N.........NL...............R.. .........MS.....
Tursiops         H.................. ....T.P...AV..S.P.N...........A.............F.R.. .........MS....N
Orcinus          H.................. ....T.P...AV..S.P.N...........A.............F.R.. .........MS....N
Lipotes          H.................. ....T.P...AV..S.P.N...........A.............F.R.. .....H...MS..Q..
Physeter         H.................. ..Q.T.P...AV..S.P.N...........A.............F.R.. ....W....MS.....
Vicugna          R.................. ....T.P....V...SP.N.........G...............F.R.. .........MS.....
Camelus          H.................. ....T.P....V...SP.N.........G...............F.R.. .......N.MS.....
Sus              ................... ....T.T....V..G.P.N..L..I...................F.R.. .........MS.....
Equus            ................... ...RA......M....PRN.........G...............F.R.. ......I..AA.....
Ceratotherium    ...R............... ....I.P....M......N.........................F.R.. ......I..AA.....
Canis            H.................. ....TL.....V....PQS..........................R.. .........MA.....
Ailuropoda       ................... ....PL.....V....PQN..N.......F...............R.. ........VMA.....
Ursus            ................... ....P......V....PQN..N.......F...............R.. ........VMA.....
Leptonychotes    H.................. ....T......V....PQN..K........P..............R.. ........MMA.....
Odobenus         H.................. ....T......V...RPQN..K........P..............R.. ........MMA.....
Mustela          H.................. ....T......M....PQS..N.......................R.. ........VMA.....
Felis            H.................. ....T.P....V....PQS..T.....................F.R.. ........MMA.....
Erinaceus        H.................. ....T.P....I.FS.P.N........T...............F.R.. .........PG.....
Sorex            R.................. ....P......S..T.P.H---......T...............F.R.. .........PS.....
Condylura        H.F................ ....TYP....V.N..P.N..T.....................F.R.. ........VPA.....
Myotis           H.................. ....P.P....V.NV.P.S..N.......................... .........MA.....
Pteropus         .....R............. ....T.P....V..T.P.S........................F.... .........MA.....
Eidolon          H.................. ....A.P....V....P.S........................F.... .........MA.....
Eptesicus        H.................. ....S.P....V.NV.P.N..N.......................R.. .........MA.....
Rhinolophus      H.................. ....T.P....V..V.P.T..N.....................F.... ........PMA.....
Pteronotus       H.................. ....T.P....V.DV.P.N..........................R.. .........MA.....
Megaderma        H.................. ....T.P....V..S.P.Q..D..P..................F.... .........MA.....
Loxodonta        H....R............. ....T.P....V..G.PQN..T.....................F.... ......A..MA.....
Procavia         H....R..R.......... ....TPP.........P.N........................F.... .........AA.....
Elephantulus     H.F..R......R...... ???????????????????????????????????????????????? .........TA.....
Echinops         H.................. ....TLP....M..L.PK...N......N.P.............F.R.. .........T......
Chrysochloris    H.................. ....TLS....V....PQT..N.....................F.R.. V........TA..Q..
Trichechus       H....R............. ....T.P....V....A.N........................F.... .....H..FMA..Q..
Sarcophilus      H....R............. ....AVTER.NV..I.P....D..I...T..............F.... .........AA.....
Monodelphis      H....R............. ....TATER.NV.NV.P.N..D..V....A.............F.... .........AA.....
Macropus         H....R..R.......... ....PVTER.NV.NV.P....D..V...T..............F.... ......A.MSA.....
```

```
                335              352          | Exon 8                                            416|
Homo            TITSSFPQLMSNVDAAYEVAERGTAYFFK GPHYWITRGFQMQGPPRTIYDFGFPRHVQQIDAAVYLREPQKTLFFVGDEYYS
Pan             ............................. .....................................................
Gorilla         ............................. .....................................................
Pongo           ............................. .............................R.......................
Nomascus        ............................. .............................R.......W...............
Papio           ............................. .............................R.......................
Mandrillus      ............................. .............................R.......................
Macaca          ............................. .....................................................
Chlorocebus     .......................I..... .............................R.......................
Rhinopithecus   ............................. .....................................................
Colobus         ............................. .....................................................
Callithrix      ............................. .................S...........R..........R............
Saimiri         ............................. ..........................Y..........................
Aotus           .................Q........... .............................R.......................
Tarsius         ............................. ..........R..................R..................E....
Otolemur        ............................. .............................R..........R............
Daubentonia     ............................. .....................................................
Microcebus      ............................. .............................R.......................
Galeopterus     ............................. ...............A.............R.......K...............
Tupaia          .......................I..... .............................R.......................
Mus             .......................I.F... .....V....H..................R.......K..........E....
Rattus          .......................I.F... .....V.......................R.......K..........E....
Cricetulus      ...................P...I.F... .............................R.......KK.........E....
Mesocricetus    .......................I.F... .................I...........R........K..............
Microtus        ...................P...L.F... .......................L..R..R........K.........E....
Jaculus         .................D.....I.F... .............................R.......K...............
Dipodomys       .......................L.F... ?????????????????????????????????????????????????????
Cavia           ............................. .....V.....I.................R.......K..R............
Octodon         ............................. ..........HI..............D..R.......K...............
Chinchilla      ............................. ...........I..............R..R.......Q...............
Fukomys         .......................V..... ...........I.................R.......K.........E.....
Heterocephalus  .......................V..... ...........I..............R..R.......KK..............
Nannospalax     ..................M....I.F... .............................R.......K...............
Ictidomys       ............................. .............................R.......KK..............
Oryctolagus     ....................D........ ............L.S...........Y..........K...............
Ochotona        ............................. .....V........S...........Y..R.....F.K...............
Bos             ............................. ..........................Y..R.......KDA.............
Capra           ....................D........ ..........................Y..........KDAR............
Ovis            ....................D........ ..........................Y..........KDA.............
Pantholops      ....................D........ ..........................Y..........KDAR............
Tursiops        ....T....I................... ..............T...........Y..R.......KDA.............
Orcinus         ............................. ..............T...........Y..R.......KDA.............
Lipotes         ............................. ..............T...........Y..R.......KDA.............
Physeter        ............................. ..............S...........Y..R.......KDA.............
Vicugna         ....................D........ .....V...............Y....N..R.......KDA.............
Camelus         ....................D........ .....V...............Y....N..R.......KDA.............
Sus             ....................D..M..... ..........................Y..R.....H.KDT.............
Equus           M...................D........ ...........I..............Y..R.....H.KDA.............
Ceratotherium   M...................D........ ..........................Y..R.......KDA.............
Canis           ............................. ..........................Y..R.......KDV.............
Ailuropoda      ....................D........ .................A........D..R.......KDV.........D...
Ursus           ....................D........ ..........................Y..R.......KDV.............
Leptonychotes   ...................SD........ ..........................Y..R.......KDV.............
Odobenus        ....................D........ ..........................Y..R.......KDV.........G...
Mustela         ....................D........ ..........................Y..R.......KDV.............
Felis           ............................. .....V....................Y..R.......KDV.............
Erinaceus       ................G............ ..........R...............Y..R.......KDA.............
Sorex           ............................. ..........................Y..R......VPDT.............
Condylura       ............................. .....T....................Y..R.......KDAE............
Myotis          ............................. ..........HI..............Y..R.......KDA.............
Pteropus        ............................. ...........I........E.....Y..R........DT.............
Eidolon         ............................. ...........I.....P........Y..R........DA.............
Eptesicus       ............................. ...........I..............Y..R.......KDA.............
Rhinolophus     ............................. ..........................Y..R.......KDA.............
Pteronotus      ............................. ..........................Y..R.......KDA.............
Megaderma       ............................. ..........................Y..R........D........A.....
Loxodonta       ........I.A............A..... .....V...........S........F..........KNA.............
Procavia        ............................. .....V...........S........F..........KDV............R
Elephantulus    .......................V..... .....V....................Y..R.......K...............
Echinops        .......................A..... .....V....................Y..RL......K.A........N....
Chrysochloris   .......................I..... .....V....................F..R.......K.A.............
Trichechus      ............................. .....V...........S.......KF..........KDA.............
Sarcophilus     ...................T.K.I.F... ........................................R.......ED...
Monodelphis     ...................T...I.F... ........................................R.......ED...
Macropus        ...................P...I.F... .....M....K..................H..........R.......ED...
```

```
                417 Exon 9                          | Exon 10                         483
Homo            YDERKRKMEKDYPKNTEEEFSGVNGQIDAAVELN GYIYFFSGPKTYKYDTEKEDVVSVVKSSSWIGC*
Pan             .....K..G......................... ..................................
Gorilla         .................................. ..................................
Pongo           ...............I.................. ..................................
Nomascus        ..............S...........V....... ..................................
Papio           .................................. ..................................
Mandrillus      .................................. ..................................
Macaca          .................................. ..................................
Chlorocebus     .................................. ..................................
Rhinopithecus   ...............I.................. ..................................
Colobus         ...............I.................. ..................................
Callithrix      ...W.............................. ..................................
Saimiri         .................................. ..................................
Aotus           .................................. ..................................
Tarsius         .................................. ..........A.......................
Otolemur        .................................. ..........A....M..............V...
Daubentonia     ........D.....S................... ..........A.......................
Microcebus      ......R................S.......... ..........A.......................
Galeopterus     ......R.......S................... ..................................
Tupaia          .................................. ..........A.............L.....V...
Mus             .....K.................S.H........ ........R..F......................
Rattus          .....K........S........S.H........ ...........F..................V...
Cricetulus      .....K........T........S..V....... ...........F..................V...
Mesocricetus    ?????????????????????????????????? ??????????????????????????????????
Microtus        .....K.................S.HV....... ...........F......................
Jaculus         F.............T........S.......... ...........F..................V...
Dipodomys       ..............S................... ...........F............L.....V...
Cavia           ..............S................... ............R..I..................
Octodon         F.............S........H..V....... ...............I..................
Chinchilla      ...........H..S........H.......... ...............V..................
Fukomys         ..............S........H.......... ...............I......G...........
Heterocephalus  ..............S........H.......... ...............I..................
Nannospalax     .......................S.......... ...........F...M..............V...
Ictidomys       .......D...............S.......... .H................................
Oryctolagus     ..............SI..............M... ..........A.............M..N......
Ochotona        F..Q..........SI.................. ........................M..N......
Bos             ..............S................... ..........A.............L.........
Capra           ..............S................... ..........A.............L.........
Ovis            ..............S................... ..........A.............L.........
Pantholops      ..............S................... ..........A.............L.........
Tursiops        ..............................M... ..........A.............L.........
Orcinus         ..............................M... ..........A.............L.........
Lipotes         ..............................M... ..........A.............L.........
Physeter        .................D................ ..........A....R........L.........
Vicugna         ..............I................... .N........A.............LRAG..V...
Camelus         .................................. .N........A.............LRAG..V...
Sus             .......D.......................... ..........A.............L..N......
Equus           ..............S................... ..........A.............L.........
Ceratotherium   ..............Y................... ..........A.............L.........
Canis           .................................. ..........A.............L.........
Ailuropoda      .................................. ..........A.............L.........
Ursus           .......................S.......... ..........A.............L.........
Leptonychotes   .................................. ..........A.............L.........
Odobenus        .................................. ..........A.............L.........
Mustela         .................................. ..........A.............L.........
Felis           .....G............................ ..........A.............L.........
Erinaceus       .....G............................ ..........A.......................
Sorex           .....G..............P............. ..........A....V..................
Condylura       ..............Y................... ..........A...................V...
Myotis          .................................. ..........A.............L.........
Pteropus        .................................. ........................L.........
Eidolon         ..........................V....... ........................L.........
Eptesicus       .................................. ..........A.............L.T.......
Rhinolophus     .................................. ..........A.............L.........
Pteronotus      ....M............................. ...............I........L.........
Megaderma       .................................. ........................L.........
Loxodonta       ....G..................S.......... ..........A.............L.........
Procavia        ???????????????????????I.?........ ..........A.............I.AN......
Elephantulus    F....M.................I.......... ..V............M..................
Echinops        ...............I.................. ..........A.......................
Chrysochloris   F....G............................ ..........A....I........I.........
Trichechus      .......................S.......... ..........A.......................
Sarcophilus     ...V...........I......IT.K......V. ..L.......A....I......N...........
Monodelphis     F..V...........V......II.K......M. ..........S....I......N.M..A.....
Macropus        ...V...........I......II.N......V. ..V.......A....I......N.R........
```
