# Supplementary material for: Evolutionary Analysis Predicts Sensitive Positions of MMP20 and Validates Newly- and Previously-Identified MMP20 Mutations Causing Amelogenesis Imperfecta
Source: Front Physiol. 2017 Jun 14;8:398. doi: 10.3389/fphys.2017.00398 (PMC5469888; doi:10.3389/fphys.2017.00398)

Supplementary Figure 2. Variations on amino acid positions in 75 mammalian MMP20. Asterisks indicate positions known to lead to Amelogenesis Imperfecta when substituted (see Figure 5).

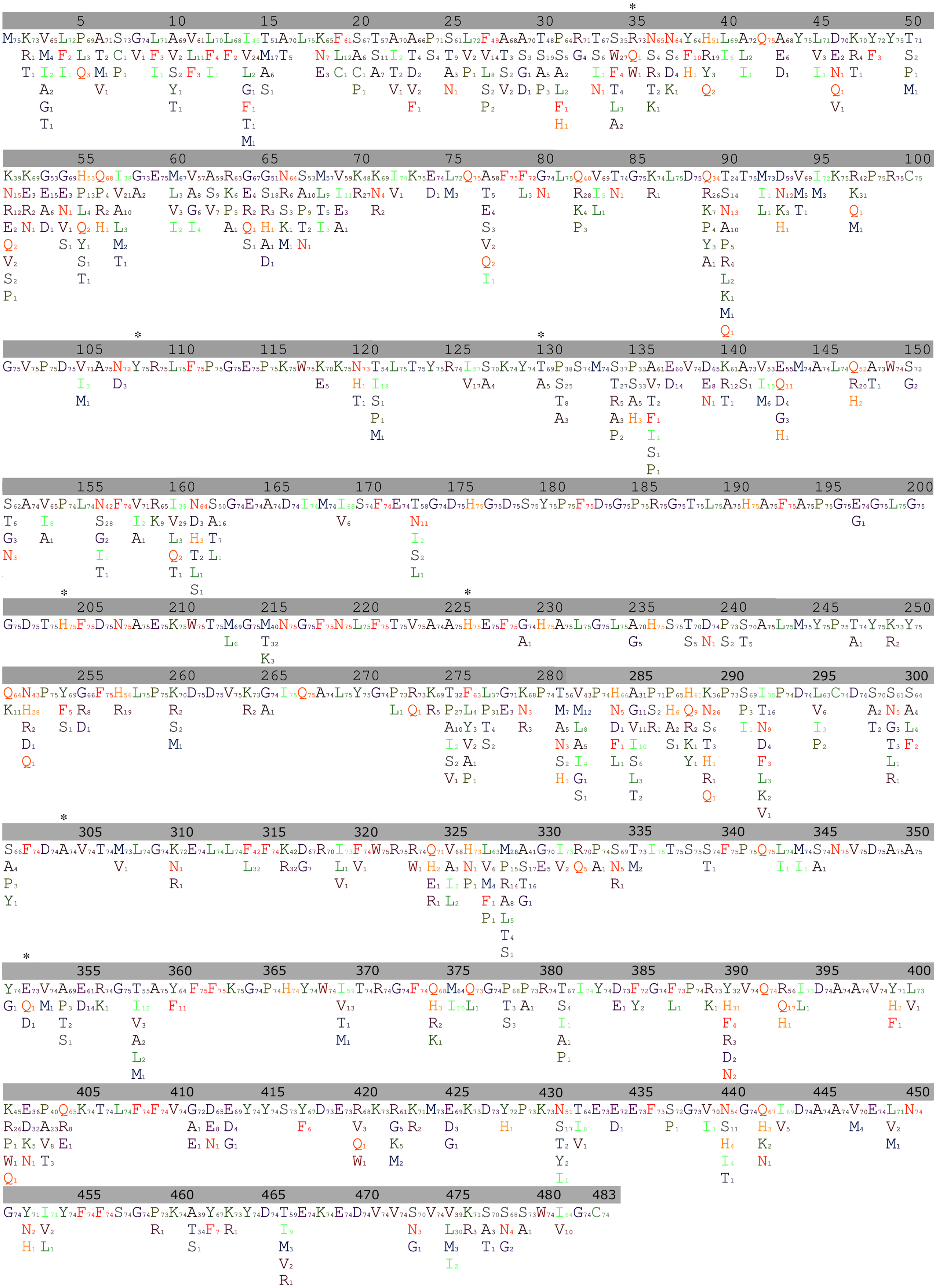

Supplement: Supplementary file 6 [file Image2.PDF]
